# Research on benefit allocation based on multi-weight H-Shapley value: A case study of express logistics sharing

**DOI:** 10.1371/journal.pone.0305656

**Published:** 2024-07-11

**Authors:** Chenglin Wang, Jintao Chen, Xiaohui Yu

**Affiliations:** School of Logistics, Beijing Wuzi University, Tongzhou District, Beijing, China; Zhengzhou University, CHINA

## Abstract

Urban last-mile express delivery in China encounters several challenges. This paper presents the establishment of a sharing logistics center aimed at enhancing the overall efficiency of urban last-mile express delivery while optimizing the utilization of essential resources. The successful implementation of shared delivery within sharing logistics center necessitates the creation of a robust collaborative mechanism. Recognizing that cooperative benefit allocation is dynamically influenced by factors such as resource input, operational efficiency, risk management, and other cost-related considerations, this study introduces a multi-weight H-Shapley value method for benefit allocation. By conducting empirical analyses of urban last-mile express delivery in Beijing within a sharing logistics service framework, our findings reveal that the revised benefit allocation model better aligns with the interests of participating entities and positively correlates with their contributions. Analyzing the impact of delivery volume and express operational costs changes, it is found that when the delivery volume and express operational costs of the sharing logistics center change, the benefits of participating enterprises move in the same direction. The benefit allocation model established in this study enriches the existing body of research in the field of shared delivery and offers valuable insights for benefit allocation issues that necessitate consideration of the dynamic effects of multiple parameter variations.

## 1. Introduction

With the development of the Internet e-commerce market and the improvement of logistics infrastructure, China’s express delivery industry has shown a leapfrog growth trend, becoming one of the fastest-growing and most vibrant emerging industries [1]. According to the State Post Bureau of the People’s Republic of China, in 2022, the global parcel delivery volume reached approximately 189.2 billion parcels, with China’s parcel delivery volume reaching 110.58 billion parcels, accounting for 58.4% of the global total. With the continuous improvement of the express delivery trunk network service system, China’s express delivery service mode is also expanding and innovating [2], the urban last-mile express delivery issue has become a focal point of concern for consumers and urban governance. Particularly in mega-cities like Beijing, various service resources within the urban areas are highly scarce, demanding constant improvement in urban governance. Therefore, the construction of a new urban last-mile express delivery service mode under sharing logistics has become a widely recognized consensus in the industry and is gradually being applied in practice with initial results. In this process, how to achieve a reasonable benefit allocation under the conditions of shared facilities, equipment, and other resources, thereby realizing efficient collaborative operations among participating entities. Especially clarifying the responsibilities between the government, enterprises, and other stakeholders, has become a key factor constraining the high-quality development of urban last-mile express delivery in sharing logistics service mode.

Due to the presence of numerous uncertain factors, such as the number of express deliveries and fluctuating operating costs, in urban last-mile express delivery of sharing logistics service mode, it is necessary to propose new solutions for benefit allocation problems that possess a certain level of fuzziness. Therefore, this study intends to utilize fuzzy numbers to represent the costs or benefits of express shared delivery in sharing logistics service mode. Subsequently, proposed an approach based on multi-weight H- Shapley value in an uncertain environment, enabling the benefit allocation in the urban last-mile express delivery of sharing logistics service mode to reflect the volatility of benefits. By analyzing the benefit allocation schemes when there are changes in express delivery volume and operating costs, the aim is to make the benefit allocation in the urban last-mile express delivery of sharing logistics service mode more scientifically rational.

## 2. Literature review

The existing research primarily explores two aspects related to last-mile express delivery and its benefit allocation: the last-mile express delivery issue based on cooperative games and research on a fuzzy benefit allocation method.

First, with regard to the problem of last-mile delivery in the context of cooperative games. Xiaohui Yu et al. [3] analyzed the issue of collaborative delivery in uncertain risk conditions and established a predictive benefit model for last-mile collaborative delivery. Daniela C et al. [4] provided an overview of supply chains from the perspective of cooperative game theory algorithms, proposing an algorithm for calculating Shapley values and validating its effectiveness through numerical examples. Xinqin Gao et al. [5] developed a cooperative optimization model based on alliance collaboration games, designed a solution process for equilibrium solutions using an improved composite method, and provided an algorithm flowchart for solving the cooperative game model. Maozeng Xu et al. [6] proposed a sharing delivery mode for express enterprises in low-density areas, analyzed the operational methods of collaborative delivery at last-mile nodes and transfer stations, and constructed a Shapley value correction mode for the transfer station sharing delivery mode. Jianjia He et al. [7] approached the issue of strategy selection and game evolution paths in the cooperation process between supply and demand enterprises by constructing a cooperative evolutionary game model from three perspectives: cooperation, competition, and neutrality. Xiaohui Yu et al. [8] analyzed the utility problem of government compensation mechanisms in solving the last-mile collaborative delivery in express logistics driven by Blockchain, and determined a reasonable range of government subsidies to express logistics enterprises and communities through evolutionary game analysis. Jinghua Zhao et al. [9] used game theory to establish two benefit allocation models: one without government subsidies and one with government subsidies. Liping Qu et al. [10] addressed the cost allocation problem in last-mile shared delivery alliances in e-commerce logistics and proposed multiple weighting approaches for resolution. Jeong-Yoo Kim [11] introduced the concept of random Shapley values, extending the definition of Shapley values to stochastic cooperative games and proving their posterior efficiency, symmetry, nullity, feasibility, and fairness. Qingbin Wang et al. [12] conducted research on the scheduling and sequencing of loading and unloading resources, establishing a non-cooperative game model for loading and unloading resource scheduling and designing a genetic algorithm for solving it. Erfang Shan et al. [13] introduced government participation to construct a multi-agent cooperative evolutionary game model and derived evolutionary stable strategies for the government and two rental markets. Sonia Mahjoub et al. [14] constructed benefit allocation rules from the duality of benefit maximization problems, and established a cooperative game model called PLSCG (Piecewise Linear Supply Chain Game) to analyze supply chain design problems. However, existing research mainly focuses on cost analysis under clear conditions, lacking studies on last-mile delivery problems with cost uncertainty.

Second, in terms of fuzzy cost allocation or benefit allocation. Jiangxia Nan et al. [15] extended fuzzy alliance cooperative games to intuitionistic fuzzy alliance cooperative games and solved the Shapley values of intuitionistic fuzzy alliance cooperative games with interval Choquet integrals. Zhengxing Zou et al. [16] studied the generalized Shapley function for cooperative games with fuzzy number payoffs based on generalized Shapley value research. Qiang Lin et al. [17] addressed the cooperative n-player strategy problem in alliances where different individuals in each coalition have different preference information, and proposed a Shapley value solution formula for fuzzy alliance value cooperative strategies based on the mean of fuzzy number preferences. Xiaohui Yu et al. [18] extended classical cooperative games and proposed a general form of fuzzy alliance cooperative games that encompasses three common types of fuzzy alliance cooperative games. Shuxia Li et al. [19] represented the payment values of cooperative strategies using triangular fuzzy numbers to solve the problem of fuzzy benefit allocation, considering the impact of alliance weights and effectiveness on benefit allocation. Zhiping Miao et al. [20] constructed a fuzzy average monotonic game model using participation degree membership functions to solve the optimal benefit allocation problem in cooperative alliances through game model solutions. Peng Guo et al. [21] introduced the credibility measure of fuzzy variables in a fuzzy game environment, established a fuzzy expected value programming model for fuzzy alliance benefit allocation, and designed a genetic algorithm to solve the proposed model, which was further demonstrated with examples. Xiaoyan Wang et al. [22] improved the interval Shapley values for fuzzy cooperative strategy benefit allocation by combining the AHP-GEM method and fuzzy comprehensive evaluation method and introducing a comprehensive correction factor for benefit allocation. Zhigang Lu et al. [23] considered the influence of various risk factors based on interval fuzzy Shapley values and used the fuzzy analytic hierarchy process to modify the interval fuzzy Shapley values, ensuring fairness in benefit allocation among supply chain participants. Ting Han et al. [24] proposed a method for solving Shapley values of intuitionistic fuzzy alliance cooperative strategies based on intuitionistic fuzzy sets, Choquet integrals, and continuous ordered weighted averaging operators, and studied the benefit allocation problem in enterprise alliances with certain participation and non-participation degrees. Hongxia Sun et al. [25] combined the Cournot model with fuzzy Shapley values of cooperative games to propose strategies for enterprise benefit allocation in cooperative alliances. Chengshou Lai et al. [26] established a triangular fuzzy number payment function and proposed fuzzy Shapley benefit allocation, introduced risk factors to modify Shapley values and solved the problem of unreasonable benefit allocation in alliance revenue uncertainty. Xiaohui Yu et al. [27] proposed an improved Shapley value method for cooperative strategies with fuzzy numbers and interval numbers to optimize cooperative allocation strategies. However, existing research mainly focuses on the benefit allocation problem in fuzzy cost-based enterprise alliances, without considering multiple factors and weights that affect benefit allocation.

In summary, previous research by scholars has primarily focused on benefit allocation in the field of express network construction and optimization, provided that benefit allocation did not yield a dynamic interval and considered fewer weights. There has been insufficient research on benefit allocation issues in the context of sharing logistics service mode, particularly in relation to cooperative modes of last-mile express delivery involving third-party logistics operators supported by the government. The current research methods mentioned do not effectively describe the issues of the research subject. The factors influencing the problem are inadequately reflected, and the proposed solutions lack dynamic characteristics, which do not align with the actual situation in the express industry. In practical scenarios, the expected benefits of each company are imprecise and fuzzy. This paper establishes a multi-weight H-Shapley value benefit allocation method for urban last-mile express delivery in sharing logistics mode. The multi-weight H-Shapley method handles cost uncertainties more effectively compared to traditional methods by incorporating triangular fuzzy numbers and extending the Shapley value to fuzzy payment cooperative strategies. The obtained benefit allocation results form a dynamic interval that can be adjusted, and considering that different enterprises have varying weights for factors influencing benefit allocation. Therefore, studying benefit allocation problems based on multi-weight triangular fuzzy numbers holds significant theoretical and practical significance.

## 3. Urban last-mile express delivery in sharing logistics service mode

### 3.1 Problem description

Currently, urban sharing logistics centers are designated by the government and operated by service providers, offering property management services. However, they do not involve equipment-sharing and information-sharing services. Within these sharing logistics centers, there are issues of poor collaboration, low operational efficiency, and unequal benefit allocation among the entities, which lack sustainability. In the actual process, benefits are allocated based on rental fees and parcel volume without considering the principles of the system or using a tiered approach for benefit allocation. The main problems are as follows.

1. There are numerous participating entities, making it difficult to achieve effective collaboration among businesses.
  The diverse range of participating entities within sharing logistics centers and the decentralized allocation of logistics resources make it challenging to achieve economies of scale at the last-mile level. While sharing logistics centers have been established, there are limited levels of information sharing among different entities, resulting in issues such as information silos, inconsistent standards, and communication barriers.
2. The high logistics costs and low operational efficiency pose challenges within the sharing logistics centers.
  Within the sharing logistics centers, there is a lack of standardization in logistics operations among brand express delivery enterprises. There is limited utilization of efficient and modern logistics equipment, and a lack of standardized operational tools. The scheduling of personnel during the last-mile delivery stage is not unified, making it challenging to meet customers’ personalized delivery demands. Additionally, the sharing logistics centers fail to effectively integrate temporal and spatial resources, hindering the achievement of intensive and efficient logistics services.
3. There is a significant disparity in the contributions of different enterprises, leading to unfair benefit allocation.
  The benefit allocation among entities within sharing logistics centers is currently conducted using traditional methods that do not consider the significant differences in initial resource investments among different entities. As a result, these methods fail to meet the diverse interests of each entity. Therefore, it is necessary to establish a benefit allocation model that takes multiple factors into account, incorporates compensatory benefits as a means of adjustment, and satisfies the interests of multiple entities.

Moreover, the volume of last-mile delivery services in Chinese cities continues to increase, yet the available urban space is becoming increasingly limited. Despite this, the average service price per delivery has not increased. The newly introduced regulations on express delivery management demand more standardized service standards. This poses greater challenges for the delivery operations of platform-based express delivery enterprises that adopt a franchising mode for last-mile delivery. As a result, delivery efficiency has declined by more than 50%, leading to a decrease in courier income. Standardization has been achieved for express delivery waybills, but the main challenge lies in the diverse management approaches among different brands. For instance, there are differences in how delivery service areas are divided, and the service standards for different levels of customers may be the same. Especially notable is the instability of operating premises, leading to operational difficulties. Both the national government and Beijing Municipality are issuing relevant policies to encourage the development of sharing logistics service modes for last-mile delivery. Specific facility and equipment configuration requirements have been proposed in Beijing’s logistics planning. There are already over 800 Cainiao stations in Beijing, making shared delivery an important component of the last-mile delivery market. The current charging standards for such stations are based on the participants’ business volume, delivery methods, and service requirements, which align closely with the methods proposed in this paper.

### 3.2 Mode design

#### 3.2.1 Urban last-mile express delivery in sharing logistics service mode

The last-mile delivery challenges in China involve multiple stakeholders, and the government’s management role is undergoing significant transformation, characterized by its dynamic nature. Existing research outcomes have primarily proposed theoretical methods for reference, but they may not entirely align with practical issues. After conducting research on the operation of shared terminal distribution, mainly in Beijing, this paper has enriched and improved the quantification of influencing factors. Additionally, it has integrated various methods used, making the research conclusions more scientifically grounded. In order to address the contradiction between the demand for specialized service resources within a region and the decentralized market entities, the government aims to increase investment. This paper proposes the establishment of a new urban sharing logistics service mode for last-mile express delivery, aiming to achieve collaboration among different brand express entities, reduce logistics costs, improve operational efficiency, and resolve the issue of benefit allocation among participating entities.

**Fig 1:**
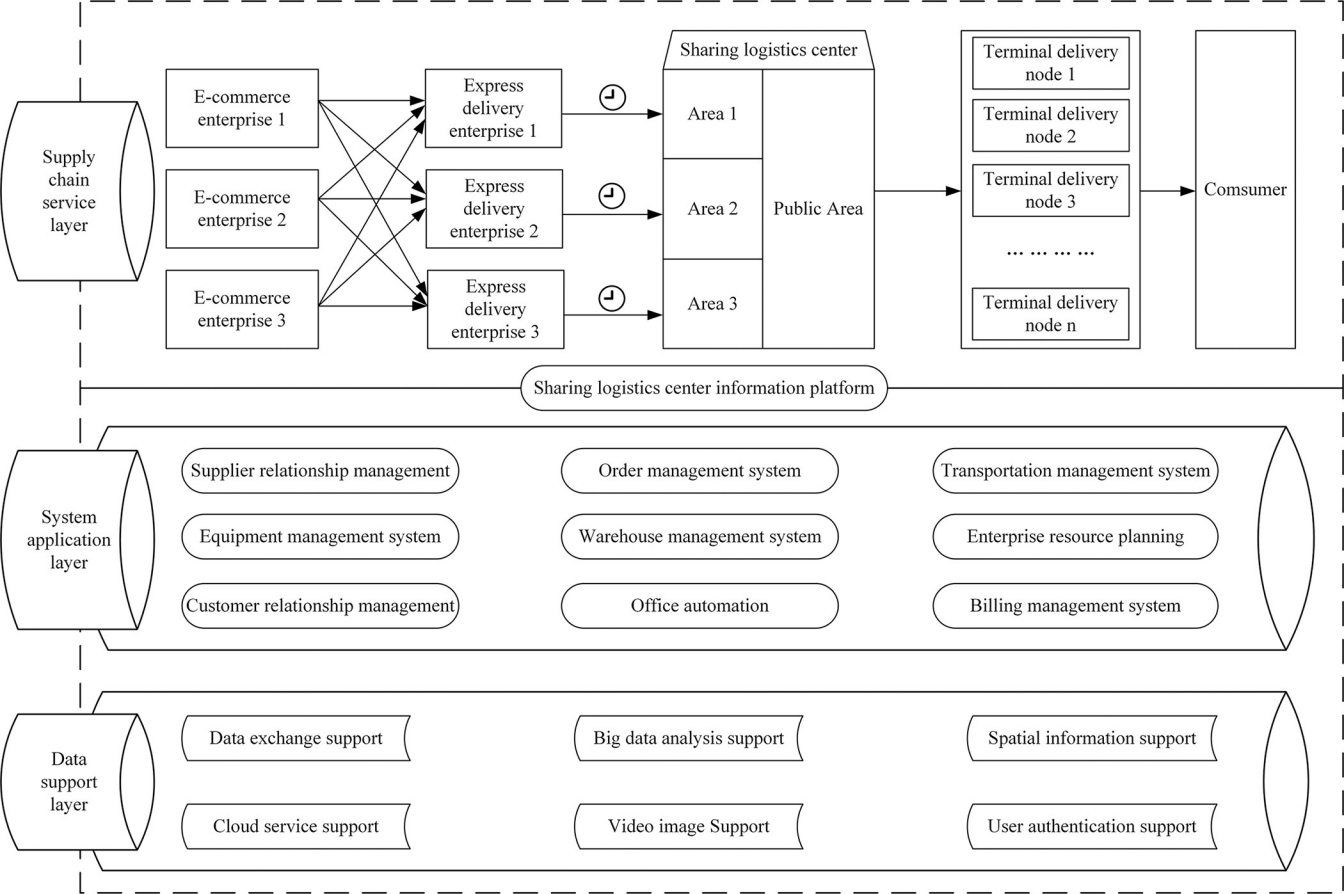
Urban last-mile express delivery in sharing logistics service mode.

The urban last-mile express delivery mode in sharing logistics service is depicted in Fig 1. The sharing logistics center serves the express delivery needs of multiple brands in the local area and can also fulfil designated responsibilities during unexpected public events, demonstrating a certain level of public welfare. Different brands of express delivery enterprises operate in the sharing logistics center through a “time-sharing” and “area-sharing” dual-sharing approach. This involves independently utilizing facilities, equipment, and other resources within the designated time frame at the center to complete sorting tasks, commence last-mile deliveries, and conduct operations such as returns and packaging within the designated brand functional areas of the center, thereby establishing unified operational norms and standards within the center. The sharing logistics center is divided into multiple functional areas, including the express vehicle waiting for area, shared operation area, express storage area, exception handling area, last-mile delivery vehicle storage area, and information control area, based on functional positioning and the type of customer services provided. This division aims to establish a standardized operational environment. During operation, the sharing logistics center offers services such as shared operation facilities, equipment, parcel reception and storage, information processing, vehicle maintenance, container packaging, packaging recycling, shared transportation capacity, and time-sharing leasing. Additionally, customized services can be provided to special delivery customers.

In this mode, express delivery enterprises can eliminate the need for infrastructure and equipment investment, allowing them to focus on their core business. Considering different express companies have varying arrival times, the operational hours are tendered on an hourly basis, operation with participating enterprises discussing the routes and vehicle actions based on the characteristics of the cosumer. In cases where there are conflicts in the operational schedules of enterprises, one potential solution is to utilize competitive bidding to determine operating times at the sharing logistics center. The government provides convenient service policies and offers public service resources for the industry through facility space planning, vehicle access, facility renovation, fund subsidies of land rental fees and other aspects. Specifically, the government provides support by offering conducive conditions, such as implementing innovative technology projects and facilitating convenient access conditions. Additionally, the government has established unified national standards for various aspects such as express waybills, address codes, and real-name systems. By standardizing address codes, it has regulated users’ address information. This helps reduce the operating costs for enterprises, creates a favorable urban governance environment, and allows the government to obtain reasonable compensatory benefits.

The operation enterprise of the sharing logistics center is coordinated through an information platform to schedule the appointment time for express delivery enterprises and analyze customer demand characteristics, thereby arranging delivery times reasonably and achieving shared delivery. This enhances the efficiency of the workspace, delivery vehicles, and personnel, thus reducing overall operating costs. Because the government has standardized express information standards, enterprises can achieve information sharing and collaboration by sharing logistics center information platforms, enabling collaborative last-mile delivery in urban express delivery under the sharing logistics mode. This platform allows real-time exchange of data, coordinates resource utilization to achieve shared delivery, and facilitates coordinated action during emergencies. The platform promotes cooperation and collaboration among enterprises, thereby enhancing the efficiency and service level of the entire urban last-mile logistics network. The New Street Express Sorting Center in the Xicheng District of Beijing serves as a worked-well example. The center covers an area of 4100 square meters, it currently hosts five express logistics companies, with a daily average sorting volume nearing fifty thousand parcels. The successful implementation of shared delivery within a sharing logistics center necessitates the creation of a robust collaborative mechanism.

#### 3.2.2 The main participants of urban last-mile express delivery in sharing logistics service mode

The urban last-mile express delivery in sharing logistics service mode based on sharing logistics center is an intensive and collaborative operational model. On the one hand, it aims to balance the risks among the key participants: express enterprises, operating enterprises, and the government. On the other hand, it seeks to establish clear responsibilities and methods for benefit allocation among these entities. The government plays a crucial role in the efficient allocation of urban last-mile express delivery sharing logistics resources by providing support in terms of land allocation and tax policies. This support is demonstrated through the determination of reasonable logistics facility space at the urban planning level and the design of sharing logistics center focused on urban last-mile, strategically planned within specific regions, and ensures a well-planned urban last-mile express delivery sharing logistics network. The government’s responsibilities mainly include supervising the operation of sharing logistics center, formulating policies and measures to promote their development, and providing public service support to improve the logistics environment and traffic conditions. Different brands of express enterprises engage in activities such as sorting, packaging, and transit delivery at upstream distribution centers. Once these operations are completed, logistics vehicles transport the packages to the sharing logistics centers, where the last-mile logistics operations continue. Express enterprises lease space and facilities in logistics centers and are responsible for the transportation, sorting, storage, and delivery of parcels. They must ensure the safety of parcels both inside and outside the sharing logistics center, comply with relevant laws and regulations, and ensure service quality and safety. Express enterprises can share operational resources within the service infrastructure based on their specific needs, thus achieving efficient microcirculation services within the region. Operating enterprises, entrusted by the government, construction and operation of the sharing logistics center. They are rewarded with reasonable returns and are not subject to specific economic performance indicators. The specific responsibilities of operating enterprises include the daily management and maintenance of the sharing logistics center, as well as the procurement of modern sorting equipment, security devices, equipment repairs, cleanliness, safety management and management information systems. They provide services such as equipment sharing and information sharing to express enterprises. They also coordinate resource utilization and provide related services such as parking management to ensure the efficient operation of the logistics center. The sharing logistics center achieves collaboration among different brand express entities, reduces logistics costs, improves operational efficiency, and the multi-weight H-Shapley method resolves the issue of benefit allocation among participating entities.

Specifically, China’s express delivery services have become an essential guarantee for people’s livelihoods, classified as social infrastructure. It is no longer merely an economic issue but also a social governance concern for the government. Currently, the shared last-mile delivery mode has become a consensus among various stakeholders, including the government and enterprises. Its essence lies in the government’s responsibility to provide planning, design, service standards, and facility guarantees, thus creating a stable operational environment. For operating and delivery companies, the core issue is to meet users’ needs while ensuring stable profits. This study comprehensively analyzes key issues such as the dynamic nature of benefits and the relationships between influencing factors, providing solutions accordingly.

## 4. Research methods

A reasonable benefit allocation method is a crucial element for urban last-mile express delivery in sharing logistics service modes. It requires the design of a quantifiable benefit allocation model based on analyzing the relationships between influencing factors.

### 4.1 Analysis of factors affecting benefit allocation

Many factors that affect the benefit allocation in the process of implementing the express delivery mode in the sharing logistics service modes. In studying the factors influencing alliance benefit allocation, Wei Chen et al. [28] introduced quantitative indicators such as investment, risk, cost, and contribution to investigate the benefit allocation in low-carbon technology innovation alliances. Deming Zeng et al. [29] considered factors such as contribution rate, input ratio, risk-bearing rate, and negotiation power to study the benefit allocation in strategic alliances for high-tech industries. Jun Li et al. [30] addressed the benefit allocation in supply chain collaborative innovation by considering factors such as innovation resource input, innovation creation benefits, and innovation risk assumption. Thus, most scholars primarily consider factors such as resource input, operational management, and risk assumption when discussing alliance benefit allocation. Based on the previous research results, through field investigation and scientific analysis of research on last-mile express logistics in Beijing, introducing diversified service factors, it can be concluded that the influencing factors of benefit allocation mainly include resource input, operational management, risk and diversified services factors.

#### 4.1.1 Resource input factors

Resource input in the context of sharing logistics center refers to the human, financial, and material resources invested in the process of conducting the business. It can be categorized into tangible assets, such as operating funds, infrastructure, operational equipment, new energy logistics vehicles, and human resources, as well as intangible assets, such as enterprise brand, professional expertise, research and innovation capabilities, and social influence. Resource input carries significant weight in the benefit allocation of sharing logistics centers. Considering that the participating members of sharing logistics services have their own strengths and operational strategies, the form and quantity of investment in sharing logistics centers may vary at different stages, thereby influencing the allocation of benefits.

1. Initial capital investment.
  When engaging in the sharing logistics and delivery business, it is crucial to have a certain level of financial support. The amount of capital investment is closely related to the smoothness of operational conditions and the future collaborative development of the business model. Particularly, it receives concentrated reflection when it comes to the allocation of benefits among the participating entities.
2. Infrastructure investment
  The application of the urban last-mile express delivery sharing logistics center cooperation mode requires specific premises, and infrastructure investment is aimed at providing support for the construction of these premises. This includes circulation processing equipment, information-sharing centers, operational sites, packaging equipment within the logistics center, and other related facilities.
3. Human resources investment.
  All employees assigned to various positions in the urban last-mile express delivery sharing logistics center cooperation mode are considered as human resources investment. From upper-level personnel such as information system maintenance and administrative management, to grassroots workers including delivery drivers and sorting personnel, they all serve as objective reflections of the service level of the logistics center. They also directly impact the final allocation of benefits and allocation costs.

#### 4.1.2 Operational management factors

The sharing logistics center can optimize the last-mile express delivery process, improve the overall operational efficiency of the system, reduce operating costs, and enhance customer satisfaction, thereby gaining more service benefits. The operational effectiveness can be evaluated using key indicators, including the number of deliveries, on-time delivery rate, loss and damage rate, green packaging rate, and the application of modern logistics technologies. Additionally, it is important to consider the impact of factors such as the level of collaboration among the cooperative entities.

1. Express delivery quantity
  The quantity of parcel deliveries is directly linked to the benefits obtained. With a higher volume of urban last-mile express delivery services, the potential for increased earnings naturally rises. In general, the more deliveries conducted by the participating enterprises in the sharing logistics alliance, the greater the benefit margin for the businesses.
2. Incidents of lost or damaged express deliveries
  During delivery operations, product damage or loss can occur due to mishandling or inadequate operational standards. By enhancing the professional skills of the operational service personnel and implementing standardized operating procedures, it is possible to minimize incidents of express package damage or loss. This trade-off of investing in operational costs aims to achieve an ideal level of logistics service while providing customers with a high-quality service experience.
3. Application of green packaging
  Prior to formal delivery, the packaging of products should be properly completed. Environmentally friendly packaging materials and reusable packaging boxes are selected to reduce the use of non-environmentally friendly packaging materials such as foam boxes and disposable bags. By doing so, in-city deliveries in Beijing, for example, can combine the advantages of low pollution, high efficiency, low consumption, high performance, and low emissions.
4. Application of logistics technology
  In transportation, the urban last-mile express delivery sharing logistics center employs traceability technology, temperature control technology, and intelligent connected vehicle systems. With the participation of these technologies, it is possible to monitor the progress and conditions of operations in real-time. However, the application of advanced logistics technology undoubtedly requires the support of related costs. Therefore, participating enterprises need to allocate additional funds on top of their original operating costs. Hence, in considering the allocation of benefits to participating enterprises, it is important to encourage greater investment in technology applications.
5. Consumer satisfaction
  The effectiveness of the urban last-mile express delivery sharing logistics center cooperation mode can be objectively reflected through consumer satisfaction. Positive consumer satisfaction motivates participating enterprises to strive for continuous improvement, expand benefit margins, and attract proactive consumer engagement. Conversely, enterprises that neglect consumer satisfaction will limit their own growth prospects. Therefore, when distributing benefits among the cooperative alliance of enterprises, the element of consumer satisfaction should be taken into consideration.

#### 4.1.3 Risk factors

Risk factors in sharing logistics center mainly include market operation coordination risk, information sharing risk, and management style differentiation risk. Many risks are specific to the shared mode, such as the risk of business data sharing and confidentiality for participating enterprises, as well as the risk of reduced service experience differentiation due to business coordination.

1. Market risk
  Currently, consumers have increasingly high expectations for product delivery in terms of product variety and quality. However, the competition among product logistics enterprises is becoming more intense, and only third-party enterprises with strong overall capabilities can establish a solid foothold in the market. Therefore, when benefits are allocated, attention should be paid to the risk-bearing capacity of each enterprise.
2. Information sharing risk
  For the urban last-mile express delivery sharing logistics center cooperation model, the shared distribution information platform is its robust software foundation. Although participating enterprises belong to the same cooperative organization, they remain independent entities. Thus, there is a possibility that they may fabricate false information or deliberately withhold information to maximize their own interests. Once such situations arise, it can lead to information asymmetry and a lack of trust among participating enterprises.
3. Ethical risks
  When engaging in cooperative management, participating enterprises should prioritize the benefits of the cooperative alliance before their own benefits. They should focus on sustainable development, and establish long-term goals, rather than solely pursuing short-term gains. When the alliance encounters cooperation problems, participating enterprises should make every effort to overcome them, work together to overcome challenges, and strive for the alliance’s substantial development.
4. Risk of management approaches differences
  Regardless of the third-party logistics enterprise, they will combine their own characteristics and learn from advanced management experiences to explore and innovate feasible management approaches. When a third-party logistics enterprise becomes a member of a cooperative organization, it may adjust its management and operational approaches accordingly. However, some individual enterprises may be unwilling to adopt new management approaches, posing significant challenges to the application of the cooperative model.

#### 4.1.4 Diversified services factors

Diversified services primarily refer to customized services for specific customer segments, providing differentiated services in terms of express delivery timeliness, delivery methods, etc. Due to the differences in development strategies and service indicators among express delivery enterprises, it is important for sharing logistics center express delivery to strike a balance between standardized services and diversified services.

### 4.2 Benefit allocation model based on multi-weight H-Shapley value method

The standard Shapley value method is widely regarded as a fair and consistent interpretation approach. Although the H-Shapley value method considers fuzziness and uncertainty, it does not take into account the consideration of weights. In contrast, the multi-weight H-Shapley value method goes a step further by incorporating the consideration of weights. This method not only considers the fuzzy relationships and uncertainty among factors but also allows for assigning different weights to different factors. Therefore, in explaining complex data and models in the real world, the multi-weight H-Shapley value method demonstrates a stronger advantage. By conducting a comparative analysis, the multi-weight H-Shapley value method can be employed to construct a benefit allocation model that meets the requirements of sharing logistics services mode.

#### 4.2.1 Fuzzy numbers and their operations

Based on the definition and basic operations of interval numbers, the following fuzzy numbers [31] and their related operations can be introduced.

**Definition 1.** If a fuzzy set $\tilde{A}$ on the real number domain R satisfies the following conditions:

1. $\tilde{A}$ is normal, which means there exists a constant *x*_0_∈*R* such that $\tilde{A}(x_{0})=1$;
2. For ∀*λ*∈(0,1], ${\tilde{A}}_{\lambda }$ is a closed interval, then $\tilde{A}$ is called a fuzzy number.

If $\mathrm{Supp}\tilde{A}$ is a finite set, then $\tilde{A}$ is a bounded fuzzy number. Based on this, if $\mathrm{Supp}\tilde{A}\subseteq [0,+\infty )$, then $\tilde{A}$ is called a non-negative fuzzy number. The set of all bounded fuzzy numbers is denoted by *FR*, and the set of all non-negative bounded fuzzy numbers is denoted by *FR*_+_.

For ∀*λ*∈(0,1], the *λ*-cut of a bounded fuzzy number $\tilde{A}$ is an interval number, represented as ${\tilde{A}}_{\lambda }=[{\tilde{A}}_{\lambda }^{-},{\tilde{A}}_{\lambda }^{+}]$. Unless otherwise stated, the fuzzy numbers in this paper are all bounded fuzzy numbers. An n-dimensional fuzzy vector refers to an n-dimensional vector where each component is a bounded fuzzy number.

The following theorem provides the necessary and sufficient condition for determining a fuzzy number using membership functions.

**Theorem 1.** [32] A fuzzy set $\tilde{A}\in FR$ exists if and only if there exist *m*,*n*∈*R* and *m*≤*n* such that:

1. On the interval [*m*,*n*], $\tilde{A}(x)=1$;
2. On the interval (−∞,*m*), $\tilde{A}(x)$ is a right-continuous increasing function, $0\leq \tilde{A}(x)<1$, $\underset{x\to -\infty }{\lim}\tilde{A}(x)=0$;
3. On the interval (*n*,+∞), $\tilde{A}(x)$ is a left-continuous decreasing function, $0\leq \tilde{A}(x)<1$, $\underset{x\to -\infty }{\lim}\tilde{A}(x)=0$.

According to Theorem 1, it is known that different membership functions determine different fuzzy numbers. Therefore, there are various types of fuzzy numbers. When representing fuzzy information for practical problems, interval numbers, triangular fuzzy numbers, and trapezoidal fuzzy numbers are commonly used. The representation of symmetric triangular fuzzy numbers is as follows:

**Definition 2.** Let $\tilde{A}=(a,b,l,r)$ be a trapezoidal fuzzy number. If *a* = *b*, that is, the membership function of $\tilde{A}$ satisfies:

$$\tilde{A}(x)=\{\begin{array}{l} \frac{x-a+l}{l},\;a-l\leq x\leq a\\ \frac{a+r-x}{r},\;a<x\leq a+r\\ 0,\;other \end{array}$$

Then $\tilde{A}$ is called a triangular fuzzy number, denoted as (*a*,*l*,*r*)_T_. Specifically, if *l* = *r*, then $\tilde{A}$ is called a symmetric triangular fuzzy number, denoted as (*a*,*l*,*l*)_T_ or (*a*,*l*)_T_, with the membership function defined as:

$$\tilde{A}(x)=\{\begin{array}{l} 1-\frac{\left|x-a\right|}{l},\;a-l\leq x\leq a+l\\ 0,\;other \end{array}$$

Furthermore, if *l* = 0, then the membership function of the symmetric triangular fuzzy number $\tilde{A}$ is defined as:

$$\tilde{A}(x)=\{\begin{array}{l} 1,\;x=a\\ 0,\;x\neq a \end{array}$$

In this case, the symmetric triangular fuzzy number $\tilde{A}$ degenerates to a real number *a*. The set of all triangular fuzzy numbers can be denoted as *R*_T_, and the set of all symmetric triangular fuzzy numbers can be denoted as *R*_ST_.

**Definition 3**. Symmetric triangular fuzzy numbers satisfy addition, subtraction, multiplication, division, and other operations. Given two symmetric triangular fuzzy numbers *β*_1_ = (*a*_0_,*b*_0_),*β*_2_ = (*a*_1_,*b*_1_), the main fuzzy arithmetic rules are as follows:

$$\beta_{1}+\beta_{2}=(a_{0},b_{0})+(a_{1},b_{1})=(a_{0}+a_{1},b_{0}+b_{1})$$

$$\beta_{1}-\beta_{2}=(a_{0},b_{0})-(a_{1},b_{1})=(a_{0}-a_{1},b_{0}+b_{1})$$

$$\beta_{1}*\beta_{2}=(a_{0},b_{0})*(a_{1},b_{1})=(a_{0}*a_{1},b_{0}*b_{1})$$

$$\beta_{1}÷\beta_{2}=(a_{0},b_{0})÷(a_{1},b_{1})=(a_{0}÷a_{1},b_{0}*b_{1})$$

**Definition 4.** Let $\tilde{A},\tilde{B}\in FR$. If there exists $\tilde{C}\in FR$, such that $\tilde{A}=\tilde{B}+\tilde{C}$, then $\tilde{C}$ is called the Hukuhara difference of $\tilde{A}$ and $\tilde{B}$, abbreviated as H-difference, denoted as $\tilde{A}{-}_{H}\tilde{B}$.

#### 4.2.2 Benefit allocation model by H-Shapley value method

Since its introduction in Shapley’s doctoral dissertation in 1953, the Shapley value [33] has garnered considerable attention in the research community. The idea behind the Shapley value method is to allocate corresponding benefits based on the ability of individuals within a coalition to contribute to the overall gains. Cooperative interval games have multiple applications in economics and operations research, where alliances formed by economic agents in cooperative relationships tend to generate higher benefits compared to those formed by independent agents [34]. Bohl et al. [35] use weighting Shapley value for market basket analysis to calculate the average revenue generated by an item. Galindo et al. [36] proposed a new solution approach using a fuzzy characteristic function, where player’s benefits are described as fuzzy numbers. They defined a value obtained from the classical Shapley value and the fuzzy number index introduced by Yager, with benefits given by real numbers. Liu et al. [37] build the Shapley allocation function, provide a more detailed and accurate picture of the fairness of the strategic contribution and reflect the degree of the players’ further choices of strategies. Zheng et al. [38] proposed a weighted Shapley value to allocate the centralized benefit, the results showed that the weighted Shapley value conditionally outperforms the classic Shapley value in the presence of fairness concerns. When cooperating and taking joint action in a coalition, it often increases the total wealth and/or decreases the total cost of the coalition [39]. In this paper, based on the definition and basic operations of interval numbers, the concept of fuzzy numbers is introduced. Similar to classical cooperative game theory, Xiaohui Yu and Qiang Zhang [40] defined axioms for the allocation of benefits using triangular fuzzy numbers, but they did not consider the issue of weights. Therefore, this paper focuses on the consideration of multi-weight issues based on the research of the former.

Sakawa and Nishizaki [41] proposed a fuzzy payment cooperative strategy by extending the payment function from real numbers to fuzzy numbers in classical cooperative game theory. Let *N* = {1,2,⋯,*n*} be the set of all players in the game. P(N) denotes the set of all subsets of N, where each element of P(N) represents a coalition. Therefore, a fuzzy payment cooperative strategy is generally defined as a binary tuple $\left(N,\tilde{v}\right)$, where the fuzzy payment function $\tilde{v}$ is a mapping from P(N) to the set of fuzzy numbers *FR*. In other words, it can be expressed as$\tilde{v}:P(N)\to FR$, satisfying $\tilde{v}(\emptyset )=0$. For the sake of convenience, this paper abbreviates the fuzzy payment cooperative strategy $\left(N,\tilde{v}\right)$ as $\tilde{v}$. In other words, $\tilde{v}$ refers to the fuzzy payment cooperative strategy *X*, where the set of players is N and the fuzzy payment function is $\tilde{v}$, $\tilde{G}(N)$ represents the set constituted by all superadditive fuzzy payment cooperative strategies $\left(N,\tilde{v}\right)$.

Suppose $\tilde{v}\in \tilde{G}(N)$, and W∈P(N). If there exists a coalition T∈P(N) such that:

$$\tilde{v}(S\cap T)=\tilde{v}(S),\;\forall S\in P(N)$$ (1)

then, *T* is referred to as the support of strategy $\tilde{v}$ in coalition *W*. Specifically, the support of strategy $\tilde{v}\in \tilde{G}(N)$ in coalition *N* is abbreviated as the support of strategy $\tilde{v}$. The set of all supports of strategy $\tilde{v}$ in the coalition *W* is denoted as $\tilde{C}(W|\tilde{v})$, defined as Eq(2):

$$\tilde{C}(W|\tilde{v})=\{T\in P(W)|\tilde{v}(S\cap T)=\tilde{v}(S),\;\forall S\in P(W)\}$$ (2)

If an n-dimensional fuzzy vector $\tilde{\varphi }(\tilde{v})=({\tilde{\varphi }}_{1}(\tilde{v}),{\tilde{\varphi }}_{2}(\tilde{v}),\ldots ,{\tilde{\varphi }}_{n}(\tilde{v}))$ satisfies the following three axioms:

**Axiom H1.** (Strong Effectiveness): If *T* is a support of strategy *v*, then $\sum_{i\in T}{\tilde{\varphi }}_{i}(\tilde{v})=\tilde{v}(S)$;

**Axiom H2.** (Symmetry): If players *i*,*j*∈*N*, and for any coalition S∈P(N\{i,j}), it holds that $\tilde{v}(S\cup \{i\})=\tilde{v}(S\cup \{j\})$, then ${\tilde{\varphi }}_{i}(\tilde{v})={\tilde{\varphi }}_{j}(\tilde{v})$.

**Axiom H3.** (Additivity): For fuzzy payment cooperative strategies $\left(N,\tilde{\mu }\right)$ and $\left(N,\tilde{\omega }\right)$, the strategy $\left(N,\tilde{\mu }+\tilde{\omega }\right)$ is defined as follows: For ∀T∈P(N), $\left(\tilde{\mu }+\tilde{\omega })(T)=\tilde{\mu }(T)+\tilde{\omega }(T\right)$, then ${\tilde{\varphi }}_{i}(\tilde{\mu }+\tilde{\omega })={\tilde{\varphi }}_{i}(\tilde{\mu })+{\tilde{\varphi }}_{i}(\tilde{\omega }),i\in N$. The resulting vector $\tilde{\varphi }(\tilde{v})$ is referred to as the Hukuhara-Shapley value of strategy $\tilde{v}$, or simply referred to as the H-Shapley value.

Based on the three axioms of the classical Shapley value, Lijiang Huang [42] proposed a cooperative payoff allocation strategy using interval and fuzzy Shapley values, defined three axioms that the fuzzy Shapley value should satisfy, and the properties of these axioms can be directly extended to the problem of benefit allocation involving triangular fuzzy numbers.

Suppose $\tilde{v}\in \tilde{G}(N)$, and if the H-Shapley value $\tilde{\varphi }(\tilde{v})$ exists and satisfies the aforementioned three axioms, then it is guaranteed to be unique and has the following form:

$${\tilde{\varphi }}_{i}(\tilde{v})=\sum_{S\in P(N\backslash \{i\})}\gamma_{S;N}[\tilde{v}(S\cup \{i\}){-}_{H}\tilde{v}(S)],\;\forall i\in N$$ (3)

for ∀*i*∈*N*, it holds that: $\gamma_{S;N}=(n-|S|-1)!|S|!/n!$, where *γ*_*S;N*_ represents the weighting factor, and $\tilde{v}(S\cup \{i\}){-}_{H}\tilde{v}(S)$ represents the H-difference between $\tilde{v}(S\cup \{i\})$ and $\tilde{v}(S)$.

#### 4.2.3 The construction of the multi-weighted H-Shapley value method for benefit allocation model

By leveraging the computational properties of fuzzy numbers, the interval Shapley is extended to incorporate fuzzy payoff cooperation strategies, and three axioms of Shapley value are expanded to construct the H-Shapley value. Furthermore, considering that different enterprises have different weights in various factors, the allocation of benefits is carried out reasonably by considering the varying contributions of participating enterprises. Due to the uncertainty associated with participants’ resource inputs, operational management, risk and diversified service factors leading to the fuzziness of alliance benefits, the proposed multi-weight H-Shapley value method for allocation addresses the problem of irrational benefit allocation in the face of uncertain alliance benefits. Moreover, it can accommodate dynamic changes in parameters such as delivery volume and operational costs.

Taking into account the characteristics of urban last-mile express delivery sharing logistics services, we consider four factors: resource input factors, operational management factors, risk factors, and diversified services factors to calculate the multi-weighted H-Shapley value. We propose a benefit allocation model based on the multi-weighted H-Shapley value method, as the following described:

$${\tilde{X}}_{i}^{'}(\tilde{\upsilon })={\tilde{X}}_{i}(\tilde{\upsilon })+\Delta {\tilde{X}}_{i}(\tilde{\upsilon })$$ (4)

In Eq (4), ${\tilde{X}}_{i}^{'}(\tilde{\upsilon })$ represents the final benefit that enterprise *i* should receive after considering multiple weights; ${\tilde{X}}_{i}(\tilde{\upsilon })$ represents the initial benefit obtained by enterprise *i*; $\Delta {\tilde{X}}_{i}(\tilde{\upsilon })$ represents the compensation value for the benefit allocation to enterprise *i*, taking into account various influencing factors by the cooperative organization; $\tilde{v}\in \tilde{G}(N)$, and its specific formula is as follows:

$$\Delta {\tilde{X}}_{i}(\tilde{\upsilon })={\tilde{X}}_{i}(\tilde{\upsilon })*\mu *\Delta \varphi_{i}$$ (5)

In Eq (5), μ (μ∈[0,1]) represents the adjustment coefficient, which is determined through research by the participating enterprises of the shared express center. Δ*φ*_*i*_ represents the overall contribution level of enterprise *i* in the shared cooperative organization.


$$\Delta \varphi_{i}=\varphi_{i}-1/n$$ (6)

In Eq (6), *φ*_*i*_ represents the contribution level of enterprise *i* to the coalition, satisfying the condition $\sum_{i=1}^{n}\varphi_{i}=1$. The term 1/*i* represents the average contribution of participating enterprises in the cooperative organization.


$$\varphi_{i}=\lambda_{i}C_{ik}$$ (7)

In Eq (7), *λ*_*i*_ represents the importance level of each influencing factor in benefit allocation, and *C*_*ik*_ represents the weight value of the *k* indicator for the *i* enterprise.

In this paper, the Analytic Hierarchy Process (AHP) [43] is used to determine the weights *λ*_*i*_ of each influencing factor in the model. The detailed process of solving the weights will be demonstrated in the subsequent example analysis.

When considering the values of the influencing factors *C*_*ik*_ in the model, it is important to note that the contribution of each participating enterprise *i* may vary for each indicator. For quantitative indicators, data can be obtained through actual surveys. However, for qualitative indicators, experts’ opinions can be gathered through questionnaire surveys. The obtained data can then be processed using Eq (8):

$$C_{ik}=C_{ik}/\sum_{i=1}^{n}C_{ik}$$ (8)

The contribution of participating enterprise *i* to the organization in the model can be calculated using Eq (9):

$$\varphi_{i}=\lambda_{i}C_{ik}$$ (9)

The overall contribution of participating enterprise *i* to the organization in the model can be calculated using Eq (10):

$$\Delta \varphi_{i}=\varphi_{i}-1/i$$ (10)

The benefit compensation value for participating enterprise *i* in the model can be calculated using Eq (11):

$$\Delta {\tilde{X}}_{i}(\tilde{\upsilon })={\tilde{X}}_{i}(\upsilon )*\mu *\Delta \varphi_{i}$$ (11)

When $\Delta {\tilde{X}}_{i}(\tilde{\upsilon })\geq 0$ represents a positive compensation value obtained by express enterprise *i*, it indicates that Δ*φ*_*i*_ is a positive value, and Δ*φ*_*i*_≥1/*i*. On the other hand, when $\Delta {\tilde{X}}_{i}(\tilde{\upsilon })\leq 0$ represents a negative compensation value obtained by participating enterprise *i*, it indicates that Δ*φ*_*i*_ is a negative value, and Δ*φ*_*i*_<1/*i*.

Therefore, the benefit allocation scheme for urban last-mile express delivery under sharing logistics based on the multi-weighted H-Shapley value method is given by: ${\tilde{X}}^{'}=({\tilde{X}}_{1}^{'}(\tilde{\upsilon }),{\tilde{X}}_{2}^{'}(\tilde{\upsilon }),{\tilde{X}}_{3}^{'}(\tilde{\upsilon }),\ldots ,{\tilde{X}}_{n}^{'}(\tilde{\upsilon }))$.

When applying H-Shapley values to the benefit allocation problem in sharing logistics services, relying solely on the marginal contribution of enterprises to the shared express organization may not provide a comprehensive solution. In practical business operations, it is necessary to consider the combined effect of various factors such as site operation costs, sorting equipment costs, labor costs, vehicle costs, and risk costs. Additionally, due to the influence of the overall market operating environment, express operation costs often fluctuate within a certain range. Therefore, the multi-weighted H-Shapley value method, combined with fuzzy number analysis, provides good applicability for analyzing the benefit allocation problem in sharing logistics center.

## 5. Case study

The Canal Business District in Beijing’s urban sub-center is located in the Tongzhou district, covering a total area of approximately 35,000 square kilometers and a construction scale of about 4.57 million square meters. It is planned as a large-scale high-end commercial district that includes industries such as high-end cultural business, exhibition offices, and entertainment and leisure. In the future, around 400,000 people will work and live in the area. The area is divided into 16 plots by developers and a domestic first large urban circular tunnel is constructed to connect the district’s underground transportation, with no vehicles allowed on the ground. Therefore, higher requirements are proposed for the express logistics service capability within the area. The Administrative Committee of the Business District allocated special facility space through government guidance and enterprise-led approach, and purchased automatic sorting equipment and other shared services for last-mile delivery pilots. A professional enterprise is responsible for the operation, while also collaborating with the Beijing Wuzi University team, who serve as technical support. This paper focuses on the practical operational situation and selects two express delivery enterprises and an operating enterprise as the main entities for benefit allocation (referred to respectively as Enterprise A, Enterprise B, and Enterprise C). Enterprise A has a daily dispatch volume of 3200 items in the area, and Enterprise B has 4300 items. Enterprise A and B can assist in terms of relevant last-mile logistics service resources, delivery vehicles, information processing, and other technical and experiential support within the area. Enterprise C is entrusted by the management institution to assume specific operations and has an experienced operational management team.

Due to the government’s strong support for its development, it has all rights to use resources such as space facilities, operating equipment, delivery vehicles, and auxiliary equipment, with operational revenue serving as a return to the Administrative Committee and other management departments. Its performance indicators require express service to meet sharing logistics delivery and other requirements and have a certain public welfare attribute. This paper selects the multi-weight H-Shapley value as the core method to address the problem of benefit allocation, considering scenarios where expected returns are represented as fuzzy numbers and the contributions of participating enterprises vary. A triangular fuzzy number payment function is constructed using fuzzy numbers, and considering that different enterprises have different weights in various factors to revise the H-Shapley benefit allocation result. Through extensive field research, foundational data such as resource input costs and operational costs were obtained. Based on past historical information, we predicted that benefits would fluctuate within a certain range. Assessing the allocation of future benefits based on such forecasts essentially involves estimating the benefits, our efforts aim to ensure the accuracy of the multi-weight H-Shapley value method to the greatest extent possible.

### 5.1 Cost and benefit identification of shared express delivery

Based on the actual operation of the sharing logistics center in this area, the primary benefit of this model is in reducing the rental costs of express delivery enterprises for venues, the redundant configuration of enterprise equipment, delivery manpower, and vehicle expenses, as well as lowering the risk costs caused by the prohibition of express delivery vehicles due to requirements for urban management improvement. Therefore, this article mainly considers resource input costs, labor costs, risk costs, and other costs when analyzing the economic benefits of the sharing logistics service mode.

#### 5.1.1 Resource input cost

Resource input costs mainly include venue costs, equipment costs, and vehicle costs. In the case of Enterprise C, it has complete ownership of all resources, including venues, equipment, and vehicles. Therefore, when cooperating with Enterprise C, there will be no venue costs, equipment costs, or vehicle costs.

1. Venue cost

The sharing logistics center mainly includes functional areas such as sorting area, office area, and parking area. Considering that third-party operating enterprises can obtain government authorization to use the venue for free, if Enterprise A and Enterprise B choose to cooperate and share the site, they can save on this cost. Based on a daily volume of 7500 parcels for both enterprises combined, the required operational area of the sorting functional zone can be calculated to be 106*m*^2^, current daily rent for facilities space in the Canal Business District is (7,0.5)_*T*_ Chinese Yuan(RMB) per square meter. The area for the other zones in the shared express center is 538*m*^2^, and the daily rental cost is (3.1,0.05)_*T*_ RMB per square meter. $\tilde{C_{1}}$ represents the annual venue cost for the sharing logistics center, and the formula for calculating the cost of the venue is shown in Eq (12), and the specific cost is listed in Table 1.


$$\tilde{C_{1}}=(S_{1}*\tilde{P_{1}}+S_{0}*\tilde{P_{0}})*365$$ (12)

*S*_1_: Area of sorting function area

$\tilde{P_{1}}$: Unit area rental cost of sorting function area

*S*_0_: Other areas of the sharing logistics center

$\tilde{P_{0}}$: Unit area rental cost of the sharing logistics center other areas

**Table 1:** Venue cost.

Enterprise	Venue cost (Unit: RMB per year)
A, B	(879577,29163.5)_*T*_
A, C	0
B, C	0
A, B, C	0

2. Equipment costs

The equipment for the sharing logistics center mainly includes the express sorting subsystem, the operation control subsystem, and other job tools. The specific procurement costs are shown in Table 2.

**Table 2:** The equipment costs for the sharing logistics center.

Equipment Details	Technical parameters	Unit	number	Unit price(Unit: 10,000 RMB)	Total Price(Unit: 10,000 RMB)
Express sorting subsystem	Number of workstations no least than 16	Set	1	(8,0)_*T*_	(8,0)_*T*_
Operation control subsystem	Data management capability for more than 10 express enterprises	Set	1	(7.5,0)_*T*_	(7.5,0)_*T*_
Other work tools	Loading and unloading, containerization equipment, etc.	Set	1	(3.79,0)_*T*_	(3.79,0)_*T*_

Assuming a 10-year service life for the equipment, the annual total maintenance cost for the equipment is estimated at 3,000 RMB. The formula for calculating the annual cost of equipment $\tilde{C_{2}}$ is shown in Eq (13), where *g* represent the type of equipment, and the specific cost is listed in Table 3.


$$\tilde{C_{2}}=(\sum_{i=1}^{g}\tilde{P}*T)/R+Y$$ (13)

$\tilde{P}$: Unit price of specification equipment

*T*: Number of specification equipment

*Y*: Total annual equipment maintenance costs

*R*: Equipment service life

**Table 3:** Equipment cost.

Enterprise	Equipment cost(Unit: RMB per year)
A, B	(22290,0)_*T*_
A, C	0
B, C	0
A, B, C	0

3. Vehicle costs

After the establishment of the sharing logistics center, each area can be equipped with dedicated facilities and equipment (including small stations and shared express cabinets) through cooperation with developers, property management, and other departments to provide delivery services for last-mile consumers. Considering the vehicle restrictions around and within the area, 4.2-meter new energy-electric trucks are used as delivery vehicles, and on average, each courier delivers 200 packages per day. The number of three-wheeled delivery vehicles is based on the principle of one vehicle per courier.

According to national regulations, the average service life of a truck is 15 years. Although there are no specific regulations for three-wheeled delivery vehicles, research shows that they are used frequently and have a relatively large cargo capacity. Therefore, a comprehensive industry experience suggests that the lifespan of trucks should be set at 10 years, while the lifespan of three-wheeled delivery vehicles should be set at 3 years, with a residual value rate of 6%. The national regulations stipulate that the energy consumption per 100 kilometers for new energy vehicles shall not exceed 10 kWh. According to the average speed of 45 km/hour and 8-hour daily operation for new energy vehicles, electricity cost is calculated based on Beijing’s industrial electricity fee of 1.1 RMB per kWh. The parameters for different specifications of vehicles are shown in Table 4, and the number of vehicles for shared express delivery according to the survey conducted by the sharing logistics center is shown in Table 5. The formula for calculating the cost of vehicle $\tilde{C_{3}}$ is shown in Eq (14), where *j* represent the type of vehicle, and the specific cost is listed in Table 6.

**Table 4:** The parameters for different specifications of vehicles.

Specification	Purchase cost (Unit: 10,000 RMB per vehicle)	Depreciation cost (Unit: RMB per month).	Fuel/Power cost (Unit: RMB per month).
Three-wheeled delivery vehicles	0.36	(95,0)_*T*_	(200,0)_*T*_
4.2-meter new energy electric trucks	10	(792,0)_*T*_	(1200,10)_*T*_

**Table 5:** Number of vehicles for sharing logistics center express delivery.

Enterprises	4.2-meter new energy electric trucks(Units: Vehicle)	Three-wheeled delivery vehicles(Units: Vehicle)
A, B	5	38
A, C	2	16
B, C	3	22
A, B, C	5	38

**Table 6:** Vehicle cost.

Enterprise	Vehicle cost(Unit: RMB per year)
A, B	(254040,5160)_*T*_
A, C	0
B, C	0
A, B, C	0


$$\tilde{C_{3}}=\sum_{i=1}^{j}V*(D+\tilde{M})*12$$ (14)

*V*: Sum of the quantity of vehicle specifications

*D*: Monthly depreciation cost of the vehicle specification

$\tilde{M}$: Monthly fuel/power cost of the vehicle specification

#### 5.1.2 Operational costs

The staff of last-mile express logistics services include couriers, sorters, drivers, and administrative personnel. According to the survey, an average courier delivers 200 packages per day. A sorter sorts one package every 9 seconds, sorting 7500 packages per day with 2 sorters arranged. Drivers are assigned according to the principle of one car per person. The number of staff for sharing logistics center express delivery is shown in Table 7. The formula for calculating labor cost ${\tilde{C}}_{4}$ is shown in Eq (15).

**Table 7:** The number of staff for after sharing logistics center express delivery.

Enterprises	Courier	Driver	Sorter	Administrative personnel
A, B	38	5	2	0
A, C	16	2	1	1
B, C	22	3	1	1
A, B, C	38	5	2	1


$${\tilde{C}}_{4}=(E_{n}*\tilde{W_{1}}+B_{n}*\tilde{W_{2}}+F_{n}*\tilde{W_{3}})*12$$ (15)

*E*_h_: The sum of the number of courier staff

$\tilde{W_{1}}$: The average salary of courier staff

*B*_*n*_: The sum number of sorter and administrative personnel staff

$\tilde{W_{2}}$: The average salary of sorter and administrative personnel staff

*F*_*n*_: The sum number of driver staff

$\tilde{W_{3}}$: The average salary of driver staff

According to the research survey, the average salary for couriers is (6000,50)_*T*_ RMB per month, the average salary for drivers is (5000,50)_*T*_ RMB per month, and the average salary for sorter and administrative personnel is (4000,50)_*T*_ RMB per month. By using the Eq (15), the labor cost can be calculated, and the specific cost is shown in Table 8.

**Table 8:** Labor costs.

Enterprises	Labor costs(Unit: RMB per year)
A, B	(3132000,27000)_*T*_
A, C	(1368000,12000)_*T*_
B, C	(1860000,16200)_*T*_
A, B, C	(3180000,27600)_*T*_

#### 5.1.3 Risk costs

According to the research survey, the risk cost of a sharing logistics center includes safety facilities costs and express insurance fees. Currently, the safety cost of a sharing logistics center is 2000 RMB per year, and the insurance fee for each package is (0.13,0.005)_*T*_ RMB per year. The formula for calculating risk cost is shown in Eq (16), and the specific cost is listed in Table 9.


$$\tilde{C_{5}}=R+\tilde{Q}*Z$$ (16)

*R*: Cost of safety facilities

$\tilde{Q}$: Insurance fee of per package

*Z*: Total amount of express delivery for per unit of time

**Table 9:** Risk costs.

Enterprises	Risk costs(Unit: RMB per year)
A, B	(357875,15687.5)_*T*_
A, C	(151840,5840)_*T*_
B, C	(204035,7847.5)_*T*_
A, B, C	(355875,13687.5)_*T*_

#### 5.1.4 Other costs

According to the research survey, other costs caused by factors such as unpredictable weather, holiday overtime, etc. account for approximately 1.3% of the total cost. Therefore, the formula for calculating other costs is as follows: Other Costs = (Sum of the Above Costs / 98.7%) * 1.3%, and the specific costs are listed in Table 10.

**Table 10:** Cost Summary (Unit: RMB per year).

Enterprises	The sum of the Above Costs	Final cost	Other costs
A, B	(4645782,77011)_*T*_	(4706973,78025)_*T*_	(61191,1014)_*T*_
A, C	(1519840,17840)_*T*_	(1539858,18075)_*T*_	(20018,235)_*T*_
B, C	(2064035,24047.5)_*T*_	(2091221,24364)_*T*_	(27186,317)_*T*_
A, B, C	(3535875,41287.5)_*T*_	(3582447,41831)_*T*_	(46572,544)_*T*_

#### 5.1.5 Shared express revenue

According to the research survey, the actual operating cost of express delivery at the end is (1.8,0.05)_*T*_ RMB per package. The benefit situation of the sharing logistics center express delivery is shown in Table 11.

**Table 11:** Benefit situation of sharing logistics center express delivery (Unit: RMB).

Enterprises	Costs before sharing logistics center express delivery	Costs after sharing logistics center express delivery.	Final revenue.
A, B	(4927500,136875)_*T*_	(4706973,78025)_*T*_	(220527,214900)_*T*_
A, C	(2102400,58400)_*T*_	(1539858,18075)_*T*_	(562542,76475)_*T*_
B, C	(2825100,78475)_*T*_	(2091221,24364)_*T*_	(733879,102839)_*T*_
A, B, C	(4927500,136875)_*T*_	(3582447,41831)_*T*_	(1345053,178706)_*T*_

If the enterprise delivers independently, it will not save any costs and therefore will not generate benefits, so $\tilde{\upsilon }(A)=\tilde{\upsilon }(B)=\tilde{\upsilon }(C)=0$. The benefit situation when Enterprise A works with B, Enterprise A works with C, Enterprise B works with C, and Enterprises A, B, and C work together are shown in Table 12.

**Table 12:** Benefits statement for each party (Unit: RMB).

A,B	A,C	B,C	A,B,C
(220527,214900)_*T*_	(562542,76475)_*T*_	(733879,102839)_*T*_	(1345053,178706)_*T*_

### 5.2 Allocation plan for benefits

Under the sharing logistics center service mode, the allocation value of the benefits of the alliance is a fuzzy situation. Since the players perceive the benefits of the alliance to be fuzzy or uncertain, then their expectations of the possible benefits allocation should also be fuzzy or uncertain. This paper compares the results obtained using the multi-weight H-Shapley value method with those from the H-Shapley value method. Through this comparison, it is demonstrated that the modified multi-weight H-Shapley value method is more in line with reality.

#### 5.2.1 Initial benefit allocation solution based on the H-Shapley value method

According to the H-Shapley solution Eq (3), the benefit values of all enterprises in the sub-strategies under the sharing logistics center express delivery mode can be obtained, and the specific benefits are shown in Table 13.

**Table 13:** Benefit values for each enterprise in all sub-strategies under the sharing logistics center express delivery mode(Unit: RMB).

$\tilde{\varphi }(\tilde{\upsilon })(W)$	*A*	*B*	*C*
$\tilde{\varphi }(\tilde{\upsilon })(\left\{A\right\})$	0	0	0
$\tilde{\varphi }(\tilde{\upsilon })(\left\{B\right\})$	0	0	0
$\tilde{\varphi }(\tilde{\upsilon })(\left\{C\right\})$	0	0	0
$\tilde{\varphi }(\tilde{\upsilon })(\left\{A,B\right\})$	(110264,107450)_*T*_	(110264,107450)_*T*_	0
$\tilde{\varphi }(\tilde{\upsilon })(\left\{A,C\right\})$	(281271,38237)_*T*_	0	(281271,38237)_*T*_
$\tilde{\varphi }(\tilde{\upsilon })(\left\{B,C\right\})$	0	(366940,51420)_*T*_	(366940,51420)_*T*_
$\tilde{\varphi }(\tilde{\upsilon })(\left\{A,B,C\right\})$	(334236,142411)_*T*_	(419905,138017)_*T*_	(590912,161088)_*T*_

For ∀*S*,*T*∈*P*(*N*),*S*⊆*T*, it is obvious that ${\tilde{\varphi }}_{i}(\tilde{\upsilon })(T)\geq {\tilde{\varphi }}_{i}(\tilde{\upsilon })(S)$, *i* = A, B, C. Therefore, Enterprise A, Enterprise B, and Enterprise C will all choose the collaboration scheme that maximizes their own benefits, which is *N* = {A, B, C}. They will each receive benefits ${\tilde{\varphi }}_{1}(\tilde{\upsilon })$, ${\tilde{\varphi }}_{2}(\tilde{\upsilon })$ and ${\tilde{\varphi }}_{3}(\tilde{\upsilon })$ respectively.

#### 5.2.2 Solution of multi-weighted H-Shapley values

In the express delivery of sharing logistics center mode, each enterprise contributes differently to the organization, which in turn contributes differently to organizational benefits. Therefore, it is necessary to consider the rational allocation of resources for each enterprise. The multi-weighted H-Shapley value method for sharing logistics center express delivery benefit allocation models relies on expert evaluation, based on the four main influencing factors of sharing logistics benefit allocation. It establishes a judgment matrix to accurately calculate the relative weight coefficients, which avoids the limitations of the H-Shapley value method only emphasizing marginal contributions. Other factors that affect benefit allocation are also considered in the allocation of benefits.

By using the AHP to determine the weight *λ*_*i*_ of each factor that affects the allocation of benefits in cooperative organizations. Using the Yaahp software to construct a model of the factors affecting the allocation of benefits in community express delivery under a sharing logistics center mode. The Yaahp software is a visualization modelling and computational tool based on the principles of the AHP. It offers functionalities such as constructing hierarchical models, generating judgment matrices, sensitivity analysis and determining weights for prioritization [44]. The specific application process is as follows: Firstly, we established a hierarchical model to the influence factor of benefit allocation in express delivery under the sharing logistics center mode; Secondly, based on the computational method of group decision-making and the scoring values from each expert, determine the judgment matrix weight of the four factors that affect benefit allocation in this article; Thirdly, by inputting the judgment matrix values in Yaahp, determine the weight distribution of each influencing factor and the overall weight distribution in the benefit allocation of community express delivery under sharing logistics center mode. The specific numerical values are shown in Tables 14 and 15.

**Table 14:** Distribution of each influencing factor among different enterprises (enterprise weight).

First-level factor	Second-level factor	Enterprise A	Enterprise B	Enterprise C
Resource input factors B1	Initial capital investment *C*_11_	0.0731	0.1087	0.8182
	Infrastructure investment *C*_12_	0.1245	0.1565	0.7190
	Land occupation *C*_13_	0	0	1
	Number of new energy vehicles *C*_14_	0.1799	0.1834	0.6367
	Human resource investment *C*_15_	0.3290	0.5635	0.1075
	Corporate image and reputation *C*_16_	0.3275	0.4317	0.2408
Operational management factors B2	Express delivery quantity *C*_21_	0.4267	0.5733	0
	Incidents of lost or damaged express deliveries *C*_22_	0.1596	0.5288	0.3116
	Rate of express delivery signature *C*_23_	0.3096	0.1230	0.5674
	Application of green packaging*C*_24_	0.0934	0.2276	0.6790
	Application of logistics technology *C*_25_	0.1345	0.2405	0.6250
	Level of collaboration with partners *C*_26_	0.1210	0.3396	0.5574
	Consumer satisfaction *C*_27_	0.0963	0.2299	0.6738
Risk factors B3	Market risks *C*_31_	0.1634	0.3170	0.5196
	Information sharing risks *C*_32_	0.0744	0.2390	0.6866
	Ethical risks *C*_33_	0.1375	0.2285	0.6340
	Policy and regulatory risks *C*_34_	0.2683	0.0967	0.6350
	Risks of management approach differences *C*_35_	0.1642	0.1872	0.6486
Diversified services factors B4	One-on-one customized services *C*_41_	0.0873	0.2479	0.6648
	One-hour delivery service *C*_42_	0.1440	0.4181	0.4379

**Table 15:** The overall weight of each factor.

Name of influencing factor	Weight *λ*_*i*_	Name of influencing factor	Weight *λ*_*i*_
Initial capital investment	0.0384	Application of logistics technology	0.0326
Infrastructure investment	0.0902	Level of collaboration with partners	0.0284
Land occupation	0.1506	Consumer satisfaction	0.0412
Number of new energy vehicles	0.0260	Market risks	0.0180
Human resource investment	0.0464	Information sharing risks	0.0506
Corporate image and reputation	0.0246	Ethical risks	0.0746
Express delivery quantity	0.0464	Policy and regulatory risks	0.0718
Incidents of lost or damaged express deliveries	0.0260	Risks of management approach differences	0.0524
Rate of express delivery signature	0.0982	One-on-one customized services	0.0372
Application of green packaging	0.0254	One-hour delivery service	0.0372

The contribution of each enterprise to the sharing logistics center express delivery mode’s benefits is determined by the allocation of secondary evaluation indicators *C*_*ik*_ (enterprise weight) in each enterprise and the global weight of secondary evaluation indicators *λ*_*i*_. By combining Tables 14 and 15, the contribution of each enterprise’s benefits can be calculated.

The contribution degree of Enterprise A is *φ*_*A*_: *φ*_*A*_ = *C*_1*k*_*λ*_*i*_ = 0.1640;

The contribution degree of Enterprise B is *φ*_*B*_: *φ*_*B*_ = *C*_2*k*_*λ*_*i*_ = 0.2213;

The contribution degree of Enterprise C is *φ*_*C*_: *φ*_*C*_ = *C*_3*k*_*λ*_*i*_ = 0.6314.

The average contribution of each enterprise in cooperation is 1/3, the comprehensive contribution of Enterprises A, B and C can be determined as follows.


$$\Delta \varphi_{A}=\varphi_{A}-1/3=0.1640-0.3333=-0.1693;$$

$$\Delta \varphi_{B}=\varphi_{B}-1/3=0.2213-0.3333=-0.1119;$$

$$\Delta \varphi_{C}=\varphi_{C}-1/3=0.6314-0.3333=0.2981.$$

The adjustment coefficient determined through negotiation between the three cooperating enterprises is 0.2, the compensation value for Enterprises A, B, and C can be determined as follows.


$$\Delta \tilde{X_{A}}=\tilde{X}(\tilde{\upsilon })*\mu \Delta \varphi_{A}={\left(1345053,178706\right)}_{T}*0.2*(-0.1693)={\left(-45543.5,-6050.99\right)}_{T}$$

$$\Delta \tilde{X_{B}}=\tilde{X}(\tilde{\upsilon })*\mu \Delta \varphi_{B}={\left(1345053,178706\right)}_{T}*0.2*(-0.1119)={\left(-30102.3,-3999.44\right)}_{T}$$

$$\Delta \tilde{X_{C}}=\tilde{X}(\tilde{\upsilon })*\mu \Delta \varphi_{c}={\left(1345053,178706\right)}_{T}*0.2*(0.2981)={\left(80192.06,10654.45\right)}_{T}$$

Determination of the revised benefit allocation of Enterprises A, B and C as follows.


$${\tilde{X_{A}}}^{'}(\tilde{\upsilon })=\tilde{X_{A}}(\tilde{\upsilon })+\Delta \tilde{X_{A}}(\tilde{\upsilon })={\left(334236,142411\right)}_{T}+{\left(-45543.5,-6050.99\right)}_{T}={\left(288693,136360\right)}_{T}$$

$${\tilde{X_{B}}}^{'}(\tilde{\upsilon })=\tilde{X_{B}}(\tilde{\upsilon })+\Delta \tilde{X_{B}}(\tilde{\upsilon })={\left(419905,138017\right)}_{T}+{\left(-30102.3,-3999.44\right)}_{T}={\left(389803,134018\right)}_{T}$$

$${\tilde{X_{C}}}^{'}(\tilde{\upsilon })=\tilde{X_{C}}(\tilde{\upsilon })+\Delta \tilde{X_{C}}(\tilde{\upsilon })={\left(590912,161088\right)}_{T}+{\left(80192.06,10654.45\right)}_{T}={\left(671104,171742\right)}_{T}$$

### 5.3 Comparative analysis of the allocation schemes

The multi-weight H-Shapley method is derived from the H-Shapley value method by introducing weights to calculate the contributions of participants, resulting in an rational allocation of benefits among participants in the alliance. Considering the uncertainty of participants’ benefits and the varying resource inputs, operational, risk and other costs of different enterprises, the revised benefit allocation scheme better reflects the actual situation. The overall weight of the resource input factors is maximum at 0.3762, operational management factors are at 0.2982, risk factors are at 0.2674 and diversified services factors is minimum at 0.0744 indicating that the resource input factor has the greatest impact on benefit allocation. Additionally, within the resource input factor, the impact of land area and infrastructure investment on benefit allocation is significant. The weight vectors directly influence the final benefit allocation results, and unreasonable weight vectors may cause the allocation results to deviate from the participants’ expectations, leading to the withdrawal of alliance participants. Therefore, the construction of reasonable weight vectors is crucial for the stability of the alliance.

The comparison of benefit allocation results before and after the revision is shown in Table 16.

**Table 16:** The comparison of benefit allocation results before and after the revision.

Participating enterprises	${\tilde{X}}_{i}^{'}(\tilde{\upsilon })$	${\tilde{X}}_{i}(\tilde{\upsilon })$	$\Delta {\tilde{X}}_{i}(\tilde{\upsilon })$
Enterprise A	(334236,142411)_*T*_	(288693,136360)_*T*_	(−45544,−6051)_*T*_
Enterprise B	(419905,138017)_*T*_	(389803,134018)_*T*_	(−30102,−3999)_*T*_
Enterprise C	(590912,161088)_*T*_	(671104,171742)_*T*_	(80192,10654)_*T*_

**Fig 2:**
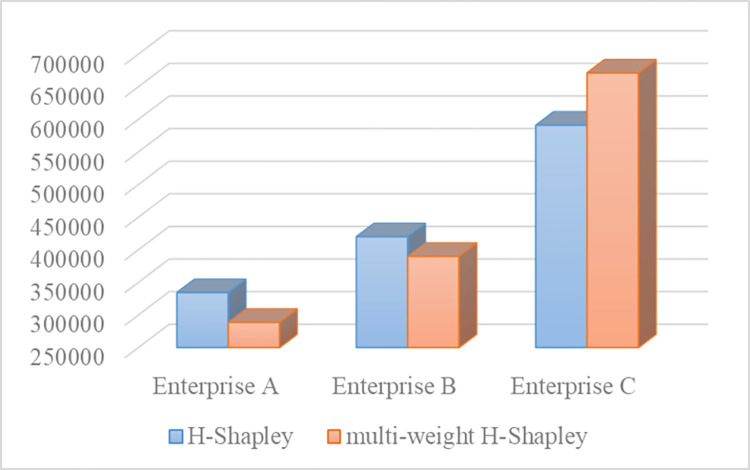
The benefit allocation results comparative of H-shapley and multi-weight H-Shapley.

According to Table 16, it can be seen that when the daily delivery volume of the shared express center is 7500 items, the three enterprises jointly developed the last-mile express sharing logistics service and gained a total benefit of (1345053,178706)_*T*_ RMB. Enterprise A, B, and C received benefits of (288693,136360)_*T*_ RMB, (389803,134018)_*T*_ RMB, and (671104,171742)_*T*_RMB, respectively. By using the benchmark values (median benefit) of each enterprise’s benefit values in Table 16 as the specific benefit, the results of H-Shapley and multi-weight H-Shapley are shown in Fig 2.

Under the initial allocation scheme, Enterprise A, B, and C have equal and each a contribution of 1/3. Under the revised scheme, the contributions of Enterprise A, B and C differ, with respective contributions of 0.1640, 0.2213 and 0.6314, and *φ*_*C*_>1/3>*φ*_*B*_>*φ*_*A*_. A comparative analysis of specific data according to Fig 2 reveals that Enterprise C receives positive compensatory benefits, while Enterprise A and B receive negative compensatory benefits. Due to the significant contribution of Enterprise C to the normal operation of the sharing logistics service mode, this research proposes transferring (80192,10654)_*T*_ RMB of income to Enterprise C, which aligns well with the actual situation where Enterprise C has invested substantial funds, facilities, and daily operations in the sharing logistics service mode. However, taking into account Enterprises A and B have lower resource inputs, Enterprise A receives (−45544,−6051)_*T*_ RMB of negative compensatory benefits and Enterprise B receives (−30102,−3999)_*T*_ RMB of negative compensatory benefits. By incorporating the varying degrees of participant contributions, refining the benefit allocation plan enhances its fairness and contributes to the stability of the cooperative organization among participating enterprises.

To sum up, this paper establishes a multi-weight H-Shapley value benefit allocation method for urban last-mile express delivery in sharing logistics mode, the benefits received by each participating enterprise in the collaborative organization of the sharing logistics service mode in the Canal Business District of Beijing’s urban sub-center need to be reasonably considered from various aspects such as the enterprise’s resource input, operational management, risk and diversified services. The obtained benefit allocation results form a dynamic interval that can be adjusted, and considered the weights of factors influencing benefit allocation. In practical situations, due to the significant resource input from Enterprise C, employing the multi-weight H-Shapley method results in Enterprise C receiving more compensatory benefits compared to the H-Shapley method.

## 6. Discussion

The express delivery market in Beijing is characterized by instability, significant fluctuations in the operating environment, and price system variations, especially in the terminal express delivery market. With the emergence of new technologies, resource input, operational, risk and other uncertainty costs are heightened. Hence, the adoption of the multi-weight H-Shapley value method is necessary for benefit allocation. The main approach involves comprehensively analyzing the influencing factors through practical operation and research, quantifying their interrelationships. Various factors constrain each other, effectively reflecting fluctuations while reducing uncertainty, thus establishing a relatively stable research context. By analyzing the impact of delivery volume and express operational costs changes, it is found that when the delivery volume and express operational costs of the sharing logistics center change, the benefits of participating enterprises move in the same direction.

### 6.1 Analysis of the impact of changes in the volume of delivery services

Under the above-mentioned unchanged basic conditions, due to the increase in the average daily delivery volume of the sharing logistics center, the concentration of the delivery area of the courier increases and the delivery efficiency per unit time is ultimately improved. As a result, the average delivery volume per person shows a linear growth trend. At the same time, considering the economic scale benefits of delivery volume and the optimal workstation limit of the sorting subsystem of the shared express center, an analysis was conducted on the interval of the daily delivery volume of the shared express center, which varied from 7,500 to 60,000 pieces. During this interval, the average daily delivery volume of the courier changed from 200 pieces to 270 pieces.

On this basis, further research was conducted on the issue of benefit allocation among Enterprise A, B, and C when the daily delivery volume of the sharing logistics center is between 7,500 and 60,000 pieces. The specific changes in benefits are shown in Table 17.

**Table 17:** Summary of benefit changes for three enterprises.

Daily delivery volume	${\tilde{X_{A}}}^{'}(\tilde{\upsilon })$	${\tilde{X_{B}}}^{'}(\tilde{\upsilon })$	${\tilde{X_{C}}}^{'}(\tilde{\upsilon })$
7500 pieces	(288693,136360)_*T*_	(389803,134018)_*T*_	(671104,171742)_*T*_
15000 pieces	(854122,266749)_*T*_	(1096980,262154)_*T*_	(1112038,331515)_*T*_
22500 pieces	(1487153,394101)_*T*_	(1910768,387310)_*T*_	(1600378,488066)_*T*_
30000 pieces	(2166943,521047)_*T*_	(2735679,511952)_*T*_	(2104030,644034)_*T*_
37500 pieces	(2914065,646577)_*T*_	(3666909,635258)_*T*_	(2653617,798251)_*T*_
45000 pieces	(3681758,771093)_*T*_	(4693333,757740)_*T*_	(3233828,951302)_*T*_
52500 pieces	(4542971,894800)_*T*_	(5742003,879195)_*T*_	(3844662,1103186)_*T*_
60000 pieces	(5424756,1017494)_*T*_	(6885869,999826)_*T*_	(4486119,1253903)_*T*_

**Fig 3:**
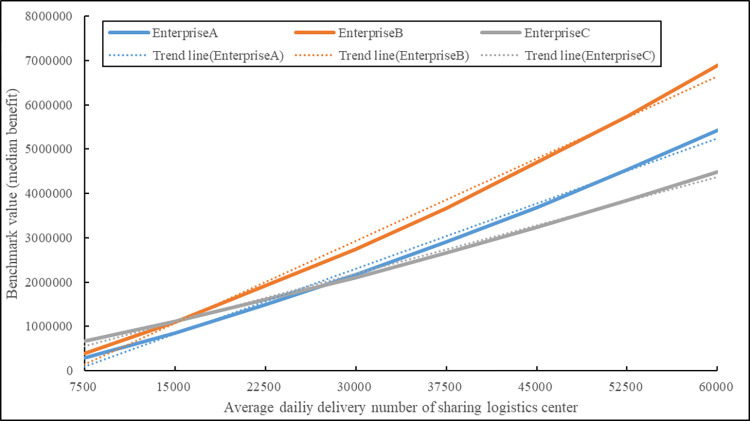
The trend chart of the change in median benefit of the enterprises as the delivery volume increases.

From Table 17, it can be seen that the adoption of this mode by the sharing logistics center will bring benefits to all participating enterprises. The key is that Enterprise A and B reduce fixed investments and venue rentals, which have lower utilization rates. On the other hand, enterprise C gathers fragmented delivery demands to form economies of scale. The benefits of Enterprise A and B primarily come from the return on investment in resources such as personnel and vehicles, while the benefits of Enterprise C mainly come from the return on investment in resources such as venue facilities and logistics equipment. Therefore, Enterprise C’s benefit is basically linearly and positively correlated with the daily delivery volume of the express. By using the benchmark values (median benefit) of each enterprise’s benefit values in Table 17 as the specific benefit, we transformed the data in the table into a line graph and performed linear fitting. The result is shown in Fig 3.

According to Fig 3, when the average daily delivery volume of A and C enterprises is around 25599 pieces, their contribution to the shared express center is the same. Therefore, their benchmark value (median benefit) is the same, at approximately 1870421 RMB. Similarly, when the average daily delivery volume of Enterprise B and C is around 15590 pieces, their benchmark value (median benefit) is the same, at approximately 1142537 RMB. The average growth rate of enterprise A is 45.99%, the average growth rate of enterprise B is 44.23%, and the average growth rate of enterprise C is 24.65%.

As the average daily delivery volume increases, the benefit growth rates of the three enterprises show differentiation. At the initial stage, Enterprise A and B have lower growth rates and receive a smaller benchmark value (median benefit) because their contribution to the overall efficiency is lower. Meanwhile, Enterprise C invests heavily in the sharing logistics center’s infrastructure, and this part of the investment has the highest cost proportion in the early stage. Thus, the contribution of Enterprise C to the overall efficiency is the highest, and it receives the highest benchmark value (median benefit). This allocation model benefits Enterprise C, which can recover its investment funds quickly. In the later stage, especially when the average daily delivery volume reaches around 30,000 pieces, the proportion of labor costs and vehicle costs will increase significantly, while the proportion of infrastructure costs will decrease sharply. Therefore, the proportion of benchmark value (median benefit) for Enterprise A and B will increase significantly, while that for Enterprise C will decrease rapidly. This allocation model will attract more express delivery enterprises to settle in the sharing logistics center in the later stage and achieve coordinated development of the economy and logistics in the Canal Business District by adjusting the express delivery mode reasonably.

### 6.2 Analysis of the impact of changes in actual operational costs of delivery services

Based on a delivery volume of 7500 pieces, the allocation of benefits among Enterprise A, B and C was studied when the actual operational cost of express last-mile delivery of the sharing logistics center is between (1.800,0.050)_*T*_ and (2.160,0.060)_*T*_ RMB per piece. The specific changes in benefits are shown in Table 18.

**Table 18:** Summary of the changes in benefits of three enterprises as the last-mile operating costs of express delivery increase.

Last-mile operating costs of express delivery	${\tilde{X_{A}}}^{'}(\tilde{\upsilon })$	${\tilde{X_{B}}}^{'}(\tilde{\upsilon })$	${\tilde{X_{C}}}^{'}(\tilde{\upsilon })$
(1.800,0.050)_*T*_	(288693,136360)_*T*_	(389803,134018)_*T*_	(671104,171742)_*T*_
(1.836,0.051)_*T*_	(455805,195358)_*T*_	(589803,185529)_*T*_	(688405,172931)_*T*_
(1.872,0.052)_*T*_	(573917,225677)_*T*_	(692743,201273)_*T*_	(700705,172929)_*T*_
(1.908,0.053)_*T*_	(652029,237351)_*T*_	(761214,205888)_*T*_	(713006,173010)_*T*_
(1.944,0.054)_*T*_	(700141,237698)_*T*_	(805684,204200)_*T*_	(725306,173164)_*T*_
(1.980,0.055)_*T*_	(738253,235226)_*T*_	(838155,200172)_*T*_	(737607,173387)_*T*_
(2.016,0.056)_*T*_	(756365,227391)_*T*_	(856625,193707)_*T*_	(749907,173672)_*T*_
(2.052,0.057)_*T*_	(774477,220706)_*T*_	(865096,185997)_*T*_	(762208,174014)_*T*_
(2.088,0.058)_*T*_	(792589,214962)_*T*_	(873566,179229)_*T*_	(774509,174409)_*T*_
(2.124,0.059)_*T*_	(810702,209997)_*T*_	(882037,173249)_*T*_	(786809,174853)_*T*_
(2.160,0.060)_*T*_	(821814,203947)_*T*_	(890507,167934)_*T*_	(799110,175342)_*T*_

**Fig 4:**
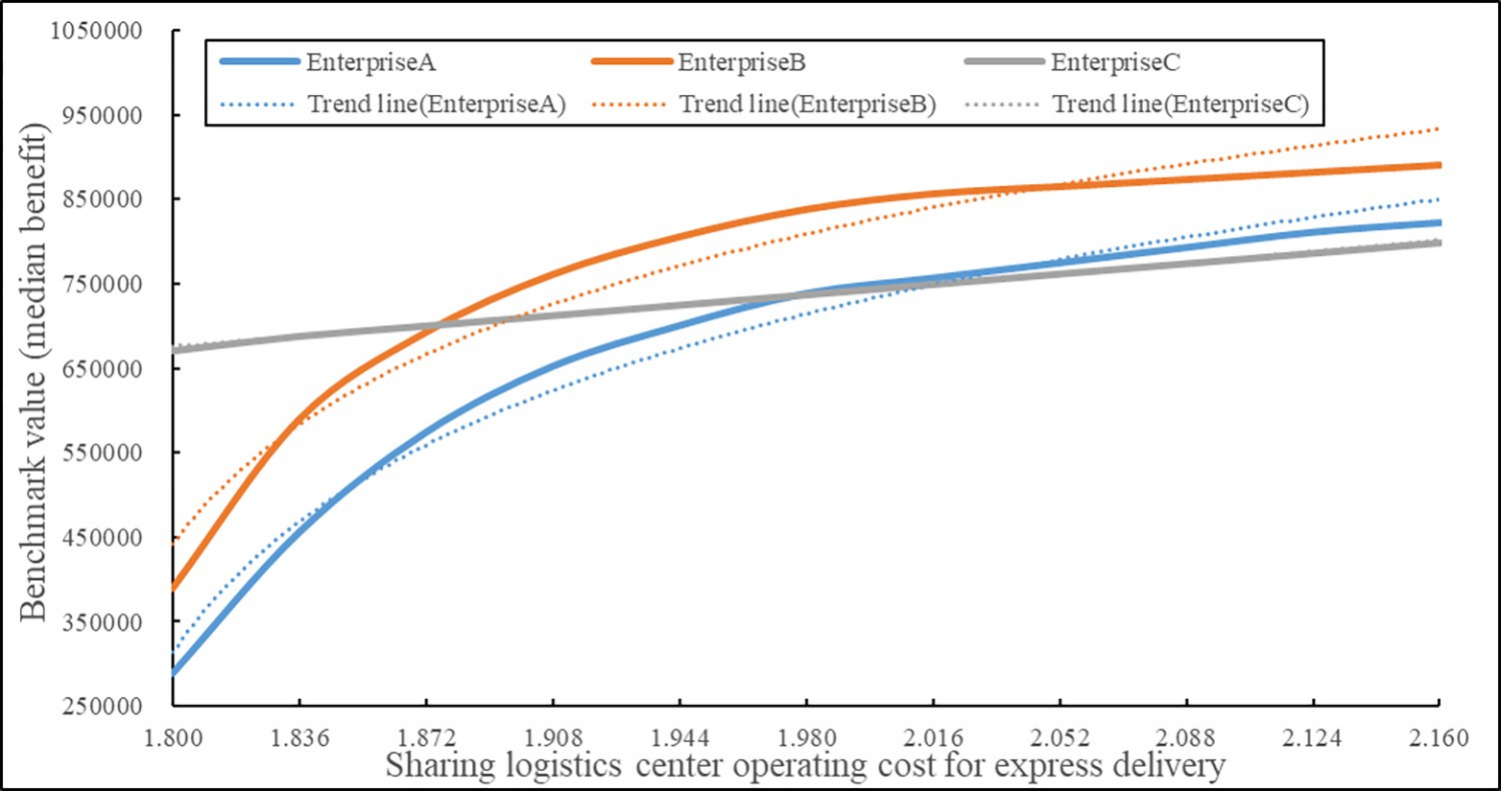
The trend of median benefit for enterprises as the operational costs of express end delivery increase.

According to Table 18, express delivery under the sharing logistics center mode, when the last-mile operating costs of express delivery increase from (1.800,0.050)_*T*_ to (2.160,0.060)_*T*_, all stakeholders can benefit from an increase in benefits. As Enterprise A and B have invested a large amount of resources such as manpower and vehicles, they can still receive a relatively large share of benefit allocation even as the delivery volume gradually decreases. Since Enterprise C does not participate in actual delivery activities, the increase in benefit is less compared to Enterprise A and B. By using the benchmark values (median benefit) figures for each enterprise in Table 18 as the actual benefits, the data in the table was transformed into a line graph and fitted linearly. The results are shown in Fig 4.

In the event of a sudden public emergency, under the sustained increase in actual operating costs of express delivery at the last-mile, the situation of benefit allocation can be observed in Fig 4. It shows that when the actual operating costs of express delivery at the last-mile increase from (1.800,0.050)_*T*_ to (1.872,0.052)_*T*_, the average growth rate of benefits for Enterprise A, B and C is 60.31%, with Enterprise A having an average growth rate of 98.80% and Enterprise B having an average growth rate of 77.72%. Customers are less sensitive to minor increases in prices, which have not had a significant impact on delivery volume. Therefore, Enterprise A and B are responsible for the actual operation and distribution of express delivery in the alliance, and their contribution to the overall alliance is relatively high. As a result, the benefits of Enterprise A and B have increased significantly. When the actual operating costs of express delivery at the last-mile increase from (1.980,0.055)_*T*_ to (2.160,0.060)_*T*_, the average growth rate of benefits for Enterprise A is 11.32%, for Enterprise B is 6.25%, and for Enterprise C is 8.34%. Since the actual operating costs of express delivery at the last-mile have increased by more than 10%, customer sensitivity is higher, resulting in a significant decrease in delivery volume, causing slower growth in overall benefits. Enterprise C invests mainly in fixed assets in the sharing logistics center, and its comprehensive contribution to the alliance tends to be stable, so it is less affected by changes in actual operating costs at the last-mile.

Based on a delivery volume of 7,500 pieces, the benefit allocation of Enterprise A, B and C was studied when the actual operating costs of express delivery at the last-mile in the sharing logistics center were in the range of (1.800,0.050)_*T*_ to (1.476,0.040)_*T*_. The specific changes in benefits are shown in Table 19.

**Table 19:** Summary table of the changes in benefits of three enterprises as the last-mile operating costs of express delivery decrease.

Last-mile operating costs of express delivery	${\tilde{X_{A}}}^{'}(\tilde{\upsilon })$	${\tilde{X_{B}}}^{'}(\tilde{\upsilon })$	${\tilde{X_{C}}}^{'}(\tilde{\upsilon })$
(1.800,0.050)_*T*_	(288693,136360)_*T*_	(389803,134018)_*T*_	(671104,171742)_*T*_
(1.764,0.049)_*T*_	(280581,148089)_*T*_	(378332,143846)_*T*_	(648804,169297)_*T*_
(1.728,0.048)_*T*_	(270469,162393)_*T*_	(365862,156133)_*T*_	(626503,166853)_*T*_
(1.692,0.047)_*T*_	(250356,175154)_*T*_	(338391,165258)_*T*_	(604203,164409)_*T*_
(1.656,0.046)_*T*_	(230244,194160)_*T*_	(309921,177823)_*T*_	(581902,161964)_*T*_
(1.620,0.045)_*T*_	(200132,214123)_*T*_	(277450,194231)_*T*_	(559601,159520)_*T*_
(1.584,0.043)_*T*_	(160020,236919)_*T*_	(234980,212849)_*T*_	(537301,157076)_*T*_
(1.548,0.042)_*T*_	(109908,269557)_*T*_	(182509,237354)_*T*_	(515000,154631)_*T*_
(1.512,0.041)_*T*_	(50796,387206)_*T*_	(120039,283912)_*T*_	(492700,152187)_*T*_
(1.476,0.040)_*T*_	(−18316,118415)_*T*_	(57569,116392)_*T*_	(470399,149742)_*T*_

**Fig 5:**
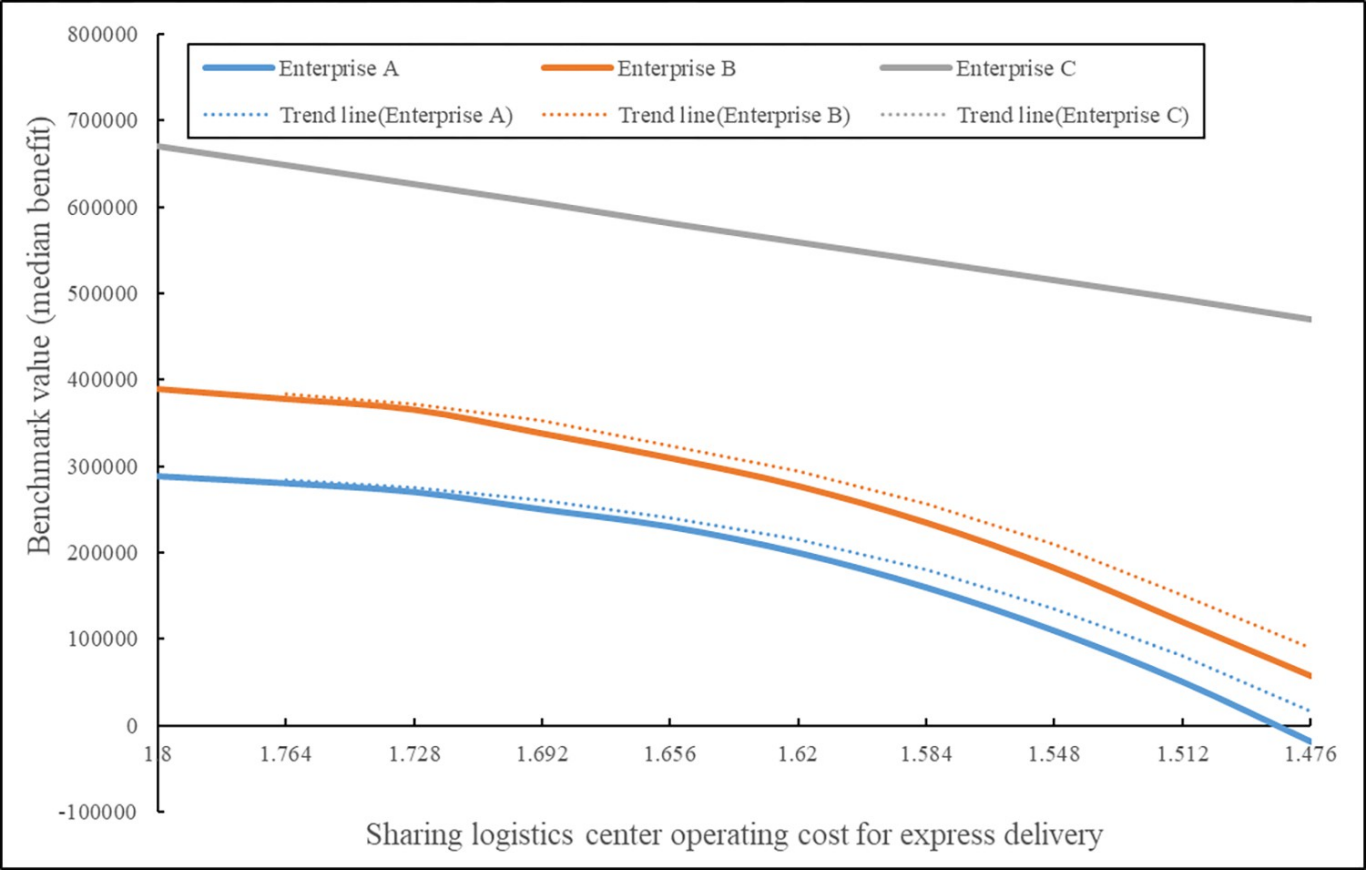
The trend of median benefit for enterprises as the operational costs of express end delivery decrease.

From Table 19, it can be observed that under the sharing logistics center mode, when the actual operating costs of express delivery at the last-mile decrease from (1.800,0.050)_*T*_ to (1.476,0.040)_*T*_ RMB, the benefits obtained by all stakeholders decrease. As Enterprise A and B invest a lot of resources such as manpower and vehicles, gain less benefit-sharing values as the delivery volume gradually increases. However, since Enterprise C primarily provides resources such as site facilities and logistics equipment, the decrease in benefits obtained is relatively lower compared to Enterprise A and B. By using the benchmark values (median benefit) figures for each enterprise in Table 19 as the specific benefits, the data in the table were transformed into a line graph and linearly fitted, as shown in Fig 5.

From Fig 5, it can be observed that in the (1.800,0.050)_*T*_ to (1.728,0.048)_*T*_ stage, the average benefit reduction rate for Enterprise A is 6.74%, for Enterprise B is 6.54%, and for Enterprise C is 7.12%. Customers are sensitive to price reductions, and with the increase in delivery business volume, Enterprises A and B, responsible for actual delivery operations and distribution in the cooperative alliance, contribute significantly to the overall alliance. Hence, the benefits of Enterprises A and B could maintain a relatively slow decline. However, in the (1.672,0.047)_*T*_ to (1.512,0.041)_*T*_ stage, the average benefit reduction rates for Enterprises A, B, and C are 300%, 492.87% and 281.90% respectively. When the delivery business volume reaches the existing delivery capacity limit, and the actual operating cost of express delivery at the last-mile continues to decline, the benefit of Enterprises A and B decreases rapidly. As a large-scale delivery enterprise, Enterprise B has strong resilience in terms of the enterprise’s resistance to pressure and supply chain. Therefore, Enterprise B reaches the critical point zero later than Enterprise A. Enterprise C mainly invests in fixed assets for the sharing logistics center, which contributes relatively stably to the overall cooperative alliance. Hence, it is less affected by changes in actual operating costs of express delivery at the last-mile. In the (1.512,0.041)_*T*_ to (1.476,0.040)_*T*_ stage, when the benefit of Enterprise A reaches zero, the express delivery alliance under sharing logistics center mode will be disbanded.

In summary, when there are changes in the volume of deliveries and operational costs at the sharing logistics center, the benefits of participating enterprises also vary accordingly. As the average daily delivery volume and operational costs change, the benefit growth rates of participating enterprises differentiate. With an increase in the average daily delivery volume, enterprises with larger resource inputs like Enterprise C experience a higher growth rate initially, followed by a gradual decrease. Enterprise A and B, due to their lower resource inputs, contribute relatively less to the overall benefits, resulting in a lower initial growth rate in benefit compared to Enterprise C, followed by a gradual increase. As Enterprise B invests more in express delivery operations than Enterprise A, its growth rate surpasses that of Enterprise A. With an increase in operational costs, enterprises A and B, which invest more in express delivery operations, have higher benchmark value benefit compared to Enterprise C, resulting in a higher growth rate for Enterprise B than Enterprise A. Since Enterprise C does not participate in express delivery operations, its income tends to stabilize. With a decrease in operational costs, the reduction in Enterprise C is relatively less compared to Enterprises A and B, which aligns with the actual situation where Enterprise C has a strong risk resistance ability and significant resource inputs. The reduction in Enterprise B’s benefit is less than that of Enterprise A, and Enterprise A’s benchmark value benefit decreases to 0 first. When the benefit of a participant reaches 0, the alliance dissolves. The multi-weight H-Shapley value method for benefit allocation can meet the dynamic changes in parameters reasonably. As the delivery volume and operational costs at the sharing logistics center increase, not only do the benefits of participating enterprises increase, but the utilization rate of express operation equipment also rises. Therefore, establishing a reasonable benefit allocation method is of significant importance for the sharing logistics service mode. Moreover, it is evident that by optimizing the allocation mechanism, the operating income of enterprises remains relatively stable. This approach can comprehensively and objectively reflect the contributions of enterprises, as changes in delivery volume and operational costs can objectively reflect their contribution levels. This is more conducive to stimulating the enthusiasm and sense of participation of enterprises, thereby creating a more stable operational environment. In cases of emergencies such as significant fluctuations in business volume, the government can formulate emergency plans for intervention to assist enterprises in resolving major issues. Additionally, providing necessary temporary support, such as equipment usage or rent reduction for premises, is essential.

## 7. Conclusion

In response to the issue of low efficiency in urban express delivery at the last-mile of the delivery chain, this article proposes an urban last-mile express delivery in sharing logistics service mode. By using cooperative game theory and establishing a multi-weight H-Shapley value benefit allocation model that considers factors such as resource input, operational management, and risk-taking, the model aims to address the issue of benefit allocation among participating entities. Taking into account the actual situation of urban last-mile demand, the model is validated through examples, and it is found that using the multi-weight H-Shapley function provides participating enterprises with more choices and leads to a more reasonable allocation of benefits. However, at present, the urban last-mile express delivery sharing logistics center service mode is only in the pilot phase with the continuous regulation of the Chinese express delivery market, and the mode is expected to be more and more successfully implemented in the future. Although the multi-weight H-Shapley value method has strong fairness [45] as a benefit allocation method, the main limitation of this study is that it lacks stability and accuracy, and it can not be adapted for different urban sizes or regulatory environments. Furthermore, how to weight the fairness and stability, accuracy of the solution will be an important subsequent research direction.

Through practical research on operational modes, it has been found that implementing sharing logistics services for last-mile delivery cannot simply rely on alliances formed by dispersed similar types of express enterprises. Instead, it requires government support for a specialized operating company to manage the operation. Currently, the allocation of benefits is a core influencing factor for increasing the enthusiasm of participants and enhancing business involvement. Factors such as business volume will also alter the allocation of benefits. Therefore, this paper integrates actual data and selects reasonable parameter ranges for quantitative research to achieve fair benefit allocation. This will effectively enhance the participation of express companies, further increasing the proportion of shared last-mile delivery, improving last-mile operational efficiency, and meeting the governance requirements of modern cities. The main conclusions drawn from this study are:

1. The multi-weight H-Shapley value method for benefit allocation can result in a more reasonable allocation of benefits. Fuzzy set analysis is highly applicable in the benefit allocation problem of shared express centers. By using the fuzzy value method to consider the differences in contributions brought about by different factors, it can meet the dynamic changes in delivery volume and operating costs and achieve a fair and reasonable allocation method.
2. Benefit allocation is a key element that affects the construction of urban last-mile express delivery under sharing logistics service mode, and the critical factors for benefit allocation are mainly influenced by factors such as resource input, operational management, and risk-taking. Among all these factors, resource input has the greatest impact on benefit allocation. Of all the factors, two factors, namely land area and infrastructure investment, have significant impacts. The government’s guidance role can be more comprehensive, focusing on early-stage cultivation. Additionally, in response to changes in business volume, the government can moderately adjust through tax policies and other measures as necessary. Furthermore, strengthening supervision of service quality and providing necessary rewards to well-performing enterprises are essential measures.
3. In the initial stages of setting up a sharing logistics center, the operating enterprise commissioned by the government invests a significant amount of resources, which rapidly increases the benchmark value (median benefit) of participating enterprises. When the delivery volume and express operational costs of the sharing logistics center change, the benefits of participating enterprises also move in the same direction. Therefore, the government can increase funding and policy support for sharing logistics centers to promote their sustainable development.

Based on the above findings, this article draws the following policy implications.

1. As the commercialization of express delivery continues to accelerate, it is crucial to properly manage the relationship between the market and enterprises, proactively establish a sharing logistics service model for urban last-mile express delivery, and regulate the market order.
2. In order to address the risks for express delivery enterprises, operation enterprises, and the government as the three main participating entities, it is important to clarify the responsibilities and allocation of benefits among them, develop reasonable plans for express delivery, and improve delivery efficiency.
3. Reasonable use of assessment and evaluation mechanisms, with a focus on addressing the issue of benefit allocation for participating enterprises, is essential to better allocate benefits and create a favorable environment for development.
